# Novel *N*-Arylaminophosphonates Bearing a Pyrrole Moiety and Their Ecotoxicological Properties

**DOI:** 10.3390/molecules22071132

**Published:** 2017-07-07

**Authors:** Jarosław Lewkowski, Marta Morawska, Anna Kaczmarek, Diana Rogacz, Piotr Rychter

**Affiliations:** 1 Department of Organic Chemistry, Faculty of Chemistry, University of Łódź, Tamka 12, 91-403 Łódź, Poland; mz.morawska@gmail.com (M.M.); ania.kaczmarek@poczta.onet.pl (A.K.); 2 Faculty of Mathematics and Natural Science, Jan Długosz University in Częstochowa, 13/15 Armii Krajowej Av., 42-200 Częstochowa, Poland; diana.rogacz@gmail.com; 3 M.Sc. Student at the Faculty of Chemistry, University of Łódź, Tamka 12, 91-403 Łódź, Poland

**Keywords:** aminophosphonates, pyrrole derivatives, aza-Pudovik reaction, ecotoxicology, *A. fischeri* test, *H. incongruens* test

## Abstract

A wide range of biological activities of aminophosphonates predisposes them to find applications as anticancer, antiviral, antimicrobial, antifungal, or herbicidal agents. Despite a number of positive aspects of the use of aminophosphonates, their applications may cause a risk to the environment, which is well exemplified by the case of glyphosate. Therefore, scientists see a pressing need to rate ecotoxicity of aminophosphonates. Nowadays, it is recommended to use comprehensive tools to carry out appropriate and effective risk assessments of toxic substances. For these purposes, tests based on the acute toxicity of the luminescent bacteria *Aliivibrio fischeri*, as well as the measurement of sub-chronic toxicity of the crustacean *Heterocypris incongruens* seem to be the most convenient. A series of five diphenyl *N*-arylamino(pyrrole-2-yl)methylphosphonates was synthesized and preliminary evaluation of their ecotoxicological properties was performed. In order to carry out such investigations, we applied the two biotests mentioned above. Results showed that the *N*-(4-nitrophenyl) derivative was the most toxic for bacteria in comparison to other tested compounds. As for crustaceans, *N*-phenyl and *N*-naphthyl derivatives were found to be the most harmful, simultaneously being relatively harmless for bacteria. Such a phenomenon are discussed in correlation with the literature, while its reason is discussed with respect to the aspect of structure of the tested compounds.

## 1. Introduction

Aminophosphonic derivatives are commonly known to have potential biological activity and this fact has been repeated and reviewed many times [1]. Nevertheless, that truism is repeated, because the area of application is very large: from pharmaceuticals, such as antibiotics or anticancer agents [2], to pesticides [1,3,4,5].

Cases of particular aminophosphonates having potential biological significance, e.g., those bearing five-membered heteroaromatic rings, have been discussed in many aspects. From among them, pyrrole-derived aminophosphonates seem to be an interesting, but unexplored, subject for studies. They were investigated with respect to their antimicrobial [6,7,8], antifungal [8], antioxidant [6,9], and anticancer activities [10], neuroactivity [11], and even anti-Alzheimer [12] action. Biological activity of these organophosphorus pyrrole derivatives is not surprising in light of numerous applications of pyrrole-derived compounds in pharmacology [13,14]

On the other hand, the nature of an ester substituent also plays an important role in the degree of biological activity of aminophosphonates. Undoubtedly, diphenyl aminophosphonates were found to be bioactive in various fields of pharmaceutical and agricultural chemistry, e.g., diphenyl aminophosphonates bearing a thiophene moiety were found to be phytotoxic for *Lepidium sativum* [15] and showed irreversible inhibiting action on chymotrypsin, human neutrophil elastase, and porcine pancreatic elastase [15]. Several tetraphenyl bisphosphonates exhibited in vitro activity in inhibition of osteoclasts growth and simultaneously demonstrated potent antioxidant activity [16]. Recent reports demonstrated antiviral activity of a series of diphenyl chiral α-aminophosphonates based on dufulin against cucumber mosaic virus [17].

Regarding the above, one should expect to find a significant number of known, reported phosphonoglycine diphenyl esters bearing a pyrrole-2-yl moiety. However, although preparation of dialkyl pyrrole-derived α-aminophosphonates are nicely represented in the chemistry literature [18,19,20,21,22], their diphenyl esters were mentioned only twice [23,24]. It is regretful, because the strong potential and usefulness of these compounds as biological agents may bring new prospects.

In order to fill the gap, we synthesized a series of diphenyl *N*-arylamino(pyrrol-2-yl)-methylphosphonates **2a**–**e**, derivatives of variously-substituted anilines and 1-naphthylamine.

Considering that REACH (Registration, Evaluation, Authorization, and Restriction of Chemicals) regulations point out to the necessity of evaluating the environmental behavior of new compounds in order to avoid the exposure on their possible harmful effects, and that such potentially bioactive compounds as α-aminophosphonic derivatives may cause environmental risks, the newly-synthesized compounds were then evaluated in this aspect.

The European Chemicals Agency (ECHA) recommended ecotoxicological testing as the essential tool for evaluating the effects of chemicals on the environment and the best way to perform that is the use of various biotests [25,26]. Toxkit microbiotests are small-scale bioassays, which are commonly used in laboratories all over the world for monitoring of contaminated waters and soils. They have many important advantages: a high degree of standardization and precision, simple testing procedures which are clearly described, small sample volumes, or the repeatability. Moreover, they give the results comparable to equivalent ISO and OECD full-scale tests. After all, the tested microorganisms are incorporated in the kits in a dormant or immobilized form, from which they can be hatched or activated “on demand” prior to carrying out the toxicity tests [27] and it makes Toxkit microbiotests much more advantageous over the conventional bioassays.

Therefore, the ecotoxicological assessment of the prepared compounds **2a**–**e** has been performed by means of two efficient biotests with *Aliivibrio fischeri* bacteria and *Heterocypris incongruens* crustaceans. In the majority of literature positions, luminescent bacteria are named *Vibrio fischeri*, but as the scientific name of the bacterium *Vibrio fischeri* has changed to *Aliivibrio fischeri* [28], we decided to use the proper nomenclature in our paper.

The Microtox^®^ system (Modern Water Inc., New Castle, DE, USA) test with the luminescent bacteria *Aliivibrio fischeri* allows the monitoring of the potential toxicity of soil or water contaminants in a simple way and to overcome disadvantages of the traditional physicochemical methods, such as the concentration of toxic substances below analytical limits or potential chemical interactions leading to additive, synergistic, or antagonistic effects which cannot be identified using the abovementioned methods. The *Aliivibrio fischeri* bioassay can be applied in all types of water, including surface and ground water, wastewater, drilling sump fluids and many other aqueous solutions [29] and has already been successfully used for ecotoxicity determination of various pharmaceuticals, pesticides, ionic liquids, detergents, heavy metals, road dust, eluates, and organic extracts of wastes, etc. [30,31,32,33,34,35,36,37]. Therefore, we decided to use the Microtox^®^ test for the preliminary evaluation of the potential toxicity of newly-synthesized aminophosphonates **2a**–**e**. Due to fact that the obtained substances are almost insoluble in water, the Microtox^®^ solid phase test (MSPT) has been used, the simplicity, reproducibility, ecological relevance, and sensitivity of which makes it one of the most popular tests for sediment toxicity assessments [32,37,38,39].

To have in hand more profound results, we have also selected the standardized crustacean biotest with *Heterocypris incongruens* (the Ostracodtoxkit^®^ test, MicroBiotests Inc, Gent, Belgium) as a representative benthic invertebrate species for screening the toxicity of sediments containing the tested substances. Crustaceans are promising representative species of the benthic environment as they live in water and in direct contact with the sediment. The Ostracodtoxkit^®^ test using the mentioned freshwater ostracodes is a sub-chronic static test that exposes individuals to whole sediments over a period of six days, the endpoints being mortality and growth changes [40].

## 2. Results

### 2.1. Preparation of Aminophosphonates ***2a**–**e***

**Scheme 1:**
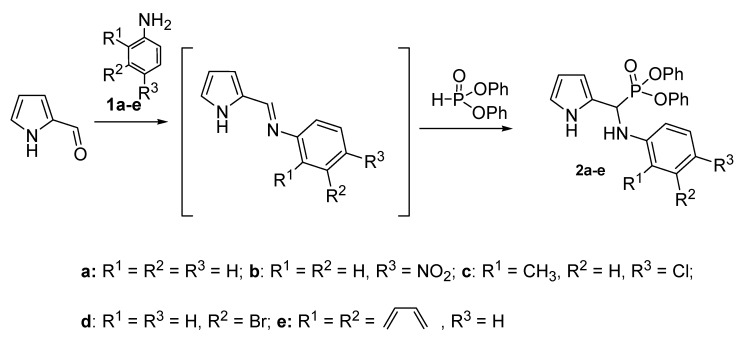
Preparation of aminophosphonates **2a**–**e**.

Synthesis of aminophosphonates **2a**–**e** was effected by means of the aza-Pudovik reaction, using the methodology determined some time ago in our laboratory. The classical method used for the aza-Pudovik reaction, i.e., preparation of a Schiff base from the mixture of an aldehyde with an amine in methanol at room temperature, its isolation and, finally, the reaction of a Schiff base with diphenyl phosphite in boiling acetonitrile or toluene [24,41,42], failed to provide the desired pyrrole-derived aminophosphonates. The methodology, which was elaborated during the realization of our previous project [43], was modified. Pyrrole-derived aminophosphonates were obtained in a two-step reaction, but Schiff bases were not isolated. (Scheme 1). The formation of Schiff bases in acetonitrile was monitored by the ^1^H NMR spectroscopy (the appearance of an azomethine proton signal at around 8.5 ppm with the simultaneous disappearance of an aldehyde proton signal at 9.5 ppm) and as soon as its formation was found complete, diphenyl phosphite was added to the mixture. The reactions were carried out for about 4 h at room temperature until precipitations formed, which were routinely isolated and determined to give aminophosphonates **2a**–**e** in fair yields (Scheme 1).

The identity of obtained products **2a**–**e** was confirmed by the ^1^H, ^13^C and ^31^P NMR spectroscopy; COSY (Correlation Spectroscopy) and Heteronuclear Multiple Quantum Correlation (HMQC) methods were used to properly assign NMR signals. ^1^H NMR spectra revealed the very interesting pattern of protons from the phenyl ring linked to a phosphonic group. It is commonly known that both ester substituents of phosphonic groups are magnetically nonequivalent, but generally signals of their particular protons lie very close to each other. In the case of diphenyl phosphonic groups, although signals of protons in positions 3 and 4 are situated very close to each other, indeed (for the *N*-phenyl derivative **2a**: H3 proton doublets of doublets at 7.33 and 7.29 ppm and triplets of H4 at 7.18 and 7.15 ppm), H2 proton doublets laid at 7.06 and 6.87 ppm, where the distance is 0.81 ppm. This pattern appeared in the spectra of all the diphenyl aminophosphonates **2b**–**e**.

^13^C NMR signals of carbon nuclei C2, C3, and C4 were found to lie very close to each other (for aminophosphonate **2a**: 130.2 and 130.1; 125.6 and 125.5; as well as doublets 121.1 and 120.9, respectively). Apart from that, expected diagnostic signals appeared, e.g., a doublet of a carbon from a P-C bond having a coupling constant around 150 Hz, at about 40 ppm.

^1^H, ^13^C and ^31^P NMR, as well as COSY and HMQC spectra of all new aminophosphonates **2a**–**e**, are collected in the Supplementary Materials.

The purity of all new compounds was verified by means of melting point measurements and elemental analysis.

### 2.2. Evaluation of Ecotoxicity of Compounds ***2a**–**e***

As it has been stated in the Introduction, ECHA suggests ecotoxicological testing of new compounds according to the REACH regulations and, therefore, we decided to follow this suggestion. The results of our previous investigations showed that aminophosphonates bearing a heteroaryl moiety, such as furfuryl [44], thien-2-yl [42], and pyrrole-2-yl [45], were ecotoxicologically harmful and the latter became the additional impulse to perform such studies.

#### 2.2.1. Ecotoxicological Effect of Compounds **2a**–**e** on *Aliivibrio fischeri* Bacteria

**Figure 1:**
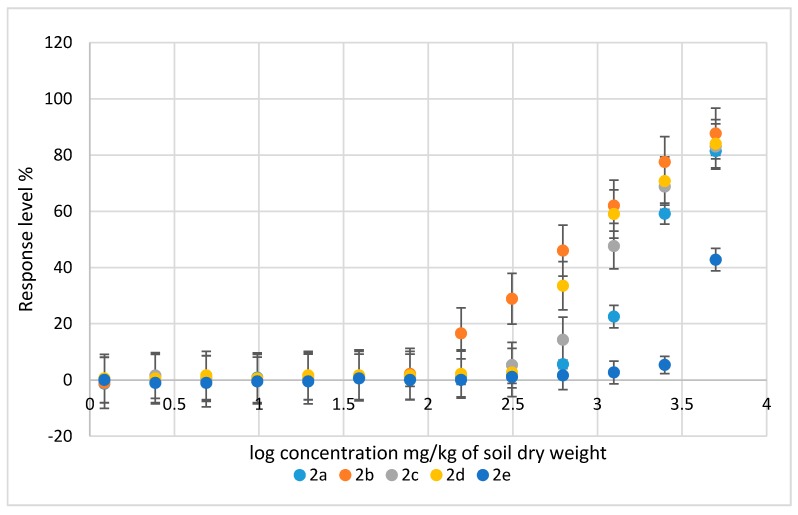
The EC_50_ graded dose-response curved for the five tested aminophosphonates **2a**–**e**. Overlapped curves are plotted by Microtox^®^ Analyzer 500 software. Error bars represent standard deviation errors (SD, *n* = 3 determinations).

Bioassays are the best tool for characterizing hazards of contaminated sediments or soils and biological effects. Light emission reduction under toxic stress of luminescent bacteria *Aliivibrio fischeri* is the basis of the applied method. In the Microtox^®^ solid phase assay, bioluminescent bacteria are brought under sediment suspensions, and their reactions fully reflected toxic effects, along with the synergistic and antagonistic agents present in the sample. The effect on light emission is assessed in the liquid phase, which remains after removal of sediment by filtration. Values of EC_50_ calculated by means of the Microtox Analyzer software are plotted in Figure 1 and presented in Table 1.

Obtained EC_50_ values for the tested compounds **2a**–**e** lay within the range between 750.9 and 5365 mg/kg of dry weight of soil. Based on the obtained results of EC_50_ values related to the ecotoxicological impact against *Aliivibrio fischeri* bacteria, the aminophosphonate **2b** should be considered as the most toxic (750.9 mg/kg of soil dry weight). Much less detrimental action against *Aliivibrio fischeri* was found for the compounds **2d** (EC_50_ = 1141 mg/kg), **2c** (EC_50_ = 1656 mg/kg), and **2a** (EC_50_ = 2262 mg/kg), while the sample containing the *N*-(1-naphthyl) derivative **2e** showed almost a lack of toxicity (EC_50_ = 5365 mg/kg).

#### 2.2.2. Ecotoxicological Impact of Compounds **2a**–**e** on *Heterocypris Incongruens* Crustaceans

**Figure 2:**
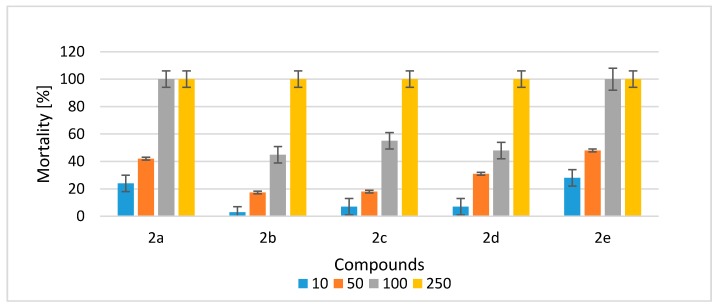
The mortality (%) of *Heterocypris incongruens* living in soil contaminated with aminophosphonates **2a**–**e** at concentrations of 10, 50, 100 and 250 mg/kg of soil dry weight. Error bars represent standard deviation error (SD, *n* = 6 determinations).

Various effects of tested substances **2a**–**e** have been shown by mortality evaluation using *Heterocypris incongruens*. The percent of morality is the first criterion of the effect on crustaceans used in tests. A growing concentration of tested substance in soil (Figure 2) resulted in, as it was expected, increased morality of ostracodes. It was found that the concentration 250 mg/kg of soil was lethal for all samples. Similar toxicity levels exhibited substances **2a** and **2e**, because the 100 mg/kg concentration, showing total mortality. Compounds **2e** demonstrated the highest toxicity at every concentration among all tested compounds when compared to other samples. Lower toxicity was exhibited by substances **2c** and **2d** to the tested organisms than **2a** and **2e**. The lowest dead impact against tested organisms was, in contrast, observed for sample **2b**.

The size of exposed ostracods living in the test sediment is compared with the dimension of ostracods living in the reference sediment at the end of the experiment, which can define the growth inhibition. It is recommended that checking growth inhibition should be determined only for sediments with a mortality of less than 30% as a criterion of sub-lethal effects to determine toxicity not elicit substantial mortality in the test organisms as selected the growth inhibition. Only when the mortality was less than 30%, the survey of lengths were performed.

In this regard, the growth inhibition rates have not been measured for aminophosphonates **2c**, **2d**, and **2b** at concentrations of 100 and 250 mg per kg of soil dry weight, and aminophosphonates **2a** and **2e** at concentrations of 50, 100 and 250 mg per kg of soil dry weight (Table 2). The effect of percentage growth inhibition of ostracods treated with growing concentrations of samples coincides with the mortality of these crustaceans. The lowest growth inhibition of ostracods was observed for sample **2b** and reached the highest concentration of only 21% when compared to untreated crustaceans. The highest growth inhibition was observed for aminophosphonates **2a** and **2e**, reaching a concentration of 10 mg/kg of soil dry weight (ca. 16%; 19%). At the upper concentration level (100 and 250 mg/kg of soil dry weight) growth inhibition, according to the Ostracodtoxkit test manual, was not calculated.

## 3. Discussion

The profitable use of bioassays for the evaluation of contamination caused by thephosphate industry wastes has already been reported and discussed [46,47]. Authors, performing the risk assessments in contaminated areas, proved that the bio-indicators based on sensitive and observable organisms constitute a very useful tool for monitoring and management of the environmental pollution. Biological analysis allows the determination of harmful effects of contaminants on organisms and provides data to reveal the mechanisms of the toxic effects.

The soil is generally the reservoir for agrochemical residues. Therefore, simple assay batteries of tests are required to control the level and decomposition of the residues of any xenobiotics, including pesticides. In the report focused on the ecotoxicological evaluation of selected pesticides (chlorpyrifos, glyphosate, vinclozolin, endosulfan), Antunes et al. have described the use of standard aquatic bioassays for the testing of soil extracts [36].

Joly et al. [34] examined the toxicity of pesticides, such as *S*-metolachlor, nicosulfuron, and benoxacor, obtaining values: 232, 218 and 121 mg/kg, respectively. Comparing the toxicity of all three herbicides with toxicities of the investigated compounds **2a**–**e**, it becomes evident that even the most toxic derivative **2b** has its EC50 value increased three to six times greater, i.e., it is three to six times less toxic for bacteria *Aliivibrio fischeri*.

The Ostracodtoxkit test using *Heterocypris incongruens* is also useful for the determination of environmental toxicity. Cvancarova et al. [48] reported the results of mortality and growth inhibition of ostracods, which correlated with various types of polycyclic aromatic hydrocarbons (PAHs). *H. incongruens* was found to be highly sensitive to bioavailable PAHs, while inhibition of *A. fischeri* luminescence varied widely between ca. 15–90% depending on the type of soil examined.

Oleszczuk and Hollert [49] reported results of studies on the influence of different soils on sewage sludge toxicity. They found that the mortality values of *Heterocypris incongruens* ranged from 0.26% up to 11.5% depending on the nature of tested sludge [49], while their growth inhibition values ranged from 10.7% to 36.2%. Płaza et al. [50] applied *H. incongruens* and *A. fischeri* to assay bioremediation processes in soils strongly contaminated with petroleum. The test species demonstrated varying sensitivity to soils and the effects on test organisms exposed to tested soils correlated with contaminant concentrations in the soil [50].

Results of toxicity against *H. incongruens* obtained for the studied aminophosphonates **2a**–**e** were at a similar level. The mortality varied from ca. 5% until ca. 30% for the concentration of 10 mg, 18–50% for the concentration 50 mg and from ca. 45% until 100% for the concentration of 100 mg per kg of soil. At higher concentrations, the tested compounds were practically lethal for crustaceans. The growth inhibition varied from 2% to 19% for a concentration of 10 mg per kg of soil.

Results of the luminescence inhibition for *Aliivibrio fischeri* tests compared with mortality and growth inhibition of crustaceans *Heterocypris incongruens* for all concentrations of studied compounds **2a**–**e** show an interesting relationship. Substances that exhibited high bacterial toxicity were not as toxic to crustaceans, and vice-versa, the lower the toxicity to *Aliivibrio fischeri*, the higher was the toxicity to *Heterocypris incongruens.* For diphenyl *N*-(4-nitrophenyl)amino-(pyrrol-2-yl)methylphosphonate (**2b**), which was the most toxic for *Aliivibrio fischeri* (EC_50_ ~750 mg/kg of soil), the lowest mortality of ostracods was revealed even at a concentration of 50 mg/kg of soil (less than 20%). It also exhibited the lowest growth inhibition degree for *H. incongruens* (21% at conc. 50 mg/kg). Such a vice versa correlation was observed for diphenyl *N*-phenylamino(pyrrol-2-yl)methylphosphonate (**2a**), which showed a high EC_50_ (2262 mg/kg of soil) value for bacteria, while for crustaceans it was the second most harmful compound in terms of mortality and inhibition of growth (~25% and 16% respectively at conc. 10 mg/kg of soil). Diphenyl *N*-(1-naphthyl)amino(pyrrol-2-yl)methylphosphonate (**2e**), which induced the lowest luminescence inhibition in *Aliivibrio fischeri* (EC_50_ = 5365), was found to be the most toxic for crustaceans considering both the mortality and the growth inhibition index (nearly 30% and 19% respectively at conc. 10 mg/kg of soil). Aminophosphonates **2c** and **2d**, which exhibited relatively moderate toxicity against *A. fischeri* were simultaneously not highly harmful for *H. incongruens*.

Such a tendency could be explained in terms of structures of amino-phosphonates **2a**–**e**. Diphenyl *N*-(4-nitrophenyl)amino(pyrrol-2-yl)methylphosphonate (**2b**), having a nitro substituent, is obviously toxic for bacteria, exactly due to the nitro group. The bactericidal action of nitro compounds is due to the action of nitroreductase on a nitro group, which leads to the formation of the active species reacting with cellular proteins [51,52]. However, its action on invertebrates, which do not have nitroreductases did not cause so much harm—the compound **2b** acted as a typical *N*-phenylamino(pyrrol-2-yl)methylphosphonate with a substituted phenyl ring. Its ecotoxicological impact on *H. incongruens* is comparable with *N*-(2-methyl-4-chlorophenyl) **2c** and *N*-(3-bromophenyl) **2d** derivatives. Diphenyl *N*-phenylamino(pyrrol-2-yl)methylphosphonate (**2a**) and diphenyl *N*-(1-naphthyl)amino(pyrrol-2-yl)methylphosphonate (**2e**), the least toxic for bacteria and the most toxic compounds for crustaceans are characterized by a not-substituted aromatic ring. A similar tendency has been described by Sihtmäe et al. [53], who studied an ecotoxicological impact of aniline and a series of substituted anilines on bacteria *Aliivibrio fischeri* and crustaceans *Daphnia magna*. The toxicity of non-substituted aniline against bacteria was evaluated as 9–30 times smaller than chloro-substituted derivatives, while its toxicological impact on crustaceans, nearly 10 times greater. It is difficult to find the reason but these findings give an impulse for further ecotoxicological studies on this class of compounds. Moreover, all the data presented above together with our previous results [41,44,45] lead us to the conclusion that *N*-arylaminophosphonic derivatives must be handled with special care, as they may be potentially hazardous for the environment.

## 4. Materials and Methods

### 4.1. Synthesis of Compounds ***2a**–**e***

#### 4.1.1. General

All solvents (POCh, Gliwice, Poland) were routinely distilled and dried prior to use. Amines, diphenyl phosphite, as well as pyrrole-2-carboxaldehyde (Aldrich, Poznań, Poland), were used as received. Melting points were measured on a MelTemp II apparatus and were not corrected. NMR spectra were recorded on a Bruker Avance III 600 MHz operating at 600 MHz (^1^H NMR), 150 MHz (^13^C NMR), and 243 MHz (^31^P NMR) or on a Varian Gemini 2000 BB operating at 81 MHz (^31^P NMR). ESI-MS were recorded using a Varian 500-MS LC ion-trap mass spectrometer (Palo Alto, CA, USA). The ESI source was operated at 5.00 kV and a capillary heater was set to 350 °C. The cone voltage was set within the range of 50–150 V. Elemental analyses were carried out at the Laboratory of Microanalysis, Faculty of Chemistry, University of Łódź, Poland.

#### 4.1.2. Preparation of Diphenyl *N*-arylamino(pyrrol-2-yl)methylphosphonates **2a**–**e** General Procedure

Pyrrole-2-carboxaldehyde (1 mmol, 0.095 g) was dissolved in acetonitrile (20 mL), and a solution of an appropriate amine (1 mmol) in acetonitrile (10 mL) was added. This solution was stirred at room temperature for 0.5 h. Then, diphenyl phosphite (1 mmol, 0.234 g) was added and the solution was stirred at room temperature. After 4 h a precipitate formed, which was collected by filtration through a fritted-glass funnel and washed with cold acetonitrile. The pure product was obtained by crystallization from ethyl acetate and hexane (1:4).

*Diphenyl N-Phenylamino(pyrrol-2-yl)methylphosphonate* (**2a**), Y = 0.331 g (82%), m.p. = 136–138 °C (white solid). ^1^H NMR (600 MHz, DMSO-*d*_6_): δ 10.84 (s, NH_pyr_, 1H); 7.33 (dd, ^3^*J*_HH_ = 7.4 and 8.6 Hz, H_Ph_, 2H); 7.29 (dd, ^3^*J*_HH_ = 7.4 and 8.6 Hz, H_Ph_, 2H); 7.18 (t, ^3^*J*_HH_ = 7.4 Hz, H_Ph_, 1H); 7.15 (t, ^3^*J*_HH_ = 7.4 Hz, H_Ph_, 1H); 7.09 (dd, ^3^*J*_HH_ = 7.2 and 8.1 Hz, H_Ph_, 2H); 7.07–7.05 (m, H_Ph_, 2H); 6.88–6.85 (m, H_Ph_, 2H); 6.80 (d, ^3^*J*_HH_ = 8.1 Hz, H_Ph_, 2H); 6.75–6.74 (m, H_pyr_, 1H); 6.62 (t, ^3^*J*_HH_ = 7.2 Hz, H_Ph_, 1H); 6.28–6.27 (m, H_pyr_, 1H); 6.23 (dd, ^3^*J*_PH_ = 3.2 and ^3^*J*_HH_ = 10.5 Hz, NH, 1H); 6.00 (dd, ^3^*J*_HH_ = 2.7 and 5.6 Hz, H_pyr_, 1H); 5.49 (dd, ^2^*J*_PH_ = 23.1 and ^3^*J*_HH_ = 10.5 Hz, CHP, 1H). ^13^C NMR (150 MHz, DMSO-*d*_6_): δ 150.7 (d, ^2^*J*_PC_ = 10.3 Hz, POC); 150.6 (d, ^2^*J*_PC_ = 10.0 Hz, POC); 147.5 (d, ^3^*J*_PC_ = 12.8 Hz, PCNC); 130.2 (C^Ph^_meta_); 130.1 (C^Ph^_meta_); 129.3 (C^Ph^_meta_); 125.6 (C^Ph^_para_); 125.5 (C^Ph^_para_); 125.2 (C^pyr^_ipso_); 121.1 (d, ^4^*J*_PC_ = 3.8 Hz, C^Ph^_ortho_); 120.9 (d, ^4^*J*_PC_ = 3.8 Hz, C^Ph^_ortho_); 118.5 (d, ^5^*J*_PC_ = 2.7 Hz, C^5^_pyr_); 118.0 (C^Ph^_para_); 114.2 (C^Ph^_ortho_); 108.6 (d, ^3^*J*_PC_ = 6.2 Hz, C^3^_pyr_); 108.3 (d, ^4^*J*_PC_ = 2.0 Hz, C^4^_pyr_); 49.5 (d, ^1^*J*_PC_ = 146.1 Hz, PC). ^31^P NMR (243 MHz, DMSO-*d*_6_): δ 15.62. IR (KBr): 3332, 3300 (νNH); 3102, 3059 (νCH_arom_); 1602, 1534, 1489, 1455 (νCC_arom_); 1228 (νP-O); 688 (∂CH_arom_). ESI MS *m/z* (%): 404 (M^+^, 73), 405 (M^+^ + 1, 21), 427 ([M + Na]^+^, 8), 443 (([M + K]^+^, 4). Elemental analysis: Calcd. for C_23_H_21_N_2_O_3_P: C, 68.31; H, 5.23; N, 6.93. Found: C, 68.10; H, 5.20; N, 7.01.

*Diphenyl N-(4-nitrophenyl)amino(pyrrol-2-yl)methylphosphonate* (**2b**), Y = 0.364 g (81%), m.p. = 161–163 °C (pale yellow powder). ^1^H NMR (600 MHz, DMSO-*d*_6_): δ 10.85 (s, NH_pyr_, 1H); 8.03 (approx d, ^3^*J*_HH_ = 9.2 Hz, CH_para_, 2H); 7.89 (dd, ^3^*J*_PH_ = 2.6 and ^3^*J*_HH_ = 9.7 Hz, NH, 1H); 7.33 (dd, ^3^*J*_HH_ = 7.4 and 8.6 Hz, H_Ph_, 2H); 7.30 (dd, ^3^*J*_HH_ = 7.3 and 8.5 Hz, H_Ph_, 2H); 7.19–7.15 (m, H_Ph_, 2H); 7.03–7.00 (m, H_Ph_, CH_para_, 4H); 6.83 (d, ^3^*J*_HH_ = 8.1 Hz, H_Ph_, 2H); 6.81–6.79 (m, H_pyr_, 1H); 6.36–6.34 (m, H_pyr_, 1H); 6.05 (dd, ^3^*J*_HH_ = 2.7 and 5.6 Hz, H_pyr_, 1H); 5.79 (dd, ^2^*J*_PH_ = 21.4 and ^3^*J*_HH_ = 9.7 Hz, CHP, 1H). ^13^C NMR (150 MHz, DMSO-*d*_6_): δ 153.6 (d, ^2^*J*_PC_ = 8.6 Hz, PCNC); 150.5 (d, ^2^*J*_PC_ = 10.1 Hz, POC); 150.4 (d, ^2^*J*_PC_ = 9.9 Hz, POC); 137.7 (CNO_2_); 130.3 (C^Ph^_meta_); 130.2 (C^Ph^_meta_); 129.3 (C^Ph^_meta_); 126.3 (C_arom_); 125.8 (C^Ph^_para_); 125.7 (C^Ph^_para_); 123.7 (C^pyr^_ipso_); 120.9 (d, ^4^*J*_PC_ = 3.8 Hz, C^Ph^_ortho_); 120.8 (d, ^4^*J*_PC_ = 3.8 Hz, C^Ph^_ortho_); 119.1 (d, ^5^*J*_PC_ = 2.4 Hz, C^5^_pyr_); 112.7 (C_arom_); 109.0 (d, ^3^*J*_PC_ = 5.3 Hz, C^3^_pyr_); 108.5 (C^4^_pyr_); 49.0 (d, ^1^*J*_PC_ = 163.4 Hz, PC). ^31^P NMR (81 MHz, DMSO-*d*_6_): δ 13.99. IR (KBr): 3332, 3294 (νNH); 3056 (νCH_arom_); 1588, 1534, 1503, 1490, 1455 (νCC_arom_); 1215 (νP-O); 836 (∂CH_arom_). ESI MS *m/z* (%): 449 (M^+^, 62), 450 (M^+^ + 1, 13), 472 ([M + Na]^+^, 4), 488 (([M + K]^+^, 3). Elemental analysis: Calcd. for C_23_H_20_N_3_O_5_P: C, 61.47; H, 4.49; N, 9.35. Found: C, 61.51; H, 4.49; N, 9.46.

*Diphenyl N-(4-chloro-2-methylphenyl)amino(pyrrol-2-yl)methylphosphonate* (**2c**), Y = 0.326 g (82%), m.p. = 152–154 °C (white crystals). ^1^H NMR (600 MHz, DMSO-*d*_6_): δ 11.02 (s, NH_pyr_, 1H); 7.34 (dd, ^3^*J*_HH_ = 7.4 and 8.5 Hz, H_Ph_, 2H); 7.29 (dd, ^3^*J*_HH_ = 7.4 and 8.6 Hz, H_Ph_, 2H); 7.19 (t, ^3^*J*_HH_ = 7.4 Hz, H_Ph_, 1H); 7.15 (t, ^3^*J*_HH_ = 7.4 Hz, H_Ph_, 1H); 7.09–7.05 (m, H_Ph_, 3H); 7.01 (dd, ^4^*J*_HH_ = 2.6 and ^3^*J*_HH_ = 8.7 Hz, H_Ph_, 1H); 6.84 (d, ^3^J_HH_ = 8.7 Hz, H_Ph_, 1H); 6.79–6.76 (m, H_Ph_, H_pyr_, 3H); 6.32–6.30 (m, H_pyr_, 1H); 6.00 (dd, ^3^*J*_HH_ = 2.8 and 5.6 Hz, H_pyr_, 1H); 5.59 (dd, ^2^*J*_PH_ = 24.7 and ^3^*J*_HH_ = 10.7 Hz, CHP, 1H); 5.11 (dd, ^3^*J*_PH_ = 3.8 and ^3^*J*_HH_ = 10.7 Hz, NH, 1H); 2.13 (s, CH_3_, 1H). ^13^C NMR (150 MHz, DMSO-*d*_6_): δ 150.8 (d, ^2^*J*_PC_ = 9.8 Hz, POC); 150.6 (d, ^2^*J*_PC_ = 10.1 Hz, POC); 143.8 (d, ^3^*J*_PC_ = 13.7 Hz, PCNC); 130.24 (C^Ph^_meta_); 130.15 (C^Ph^_meta_); 129.9 (C_Ph_); 126.5 (C_Ph_); 125.7 (C^Ph^_para_); 125.6 (C^Ph^_para_); 124.5 (C^pyr^_ipso_); 121.9 C_Ph_); 120.9 (d, ^4^*J*_PC_ = 3.7 Hz, C^Ph^_ortho_); 120.8 (d, ^4^*J*_PC_ = 3.9 Hz, C^Ph^_ortho_); 119.0 (d, ^5^*J*_PC_ = 2.7 Hz, C^5^_pyr_); 113.6 (C_Ph_); 109.1 (d, ^3^*J*_PC_ = 7.5 Hz, C^3^_pyr_); 108.2 (d, ^4^*J*_PC_ = 1.8 Hz, C^4^_pyr_); 50.1 (d, ^1^*J*_PC_ = 162.9 Hz, PC); 17.6 (CH_3_). ^31^P NMR (81 MHz, DMSO-*d*_6_): δ 14.97. IR (KBr): 3368, 3306 (νNH); 1588, 1509, 1489, 1456 (νCC_arom_); 1234 (νP-O); 799 (∂CH_arom_); 728 (∂CC_arom_). ESI MS *m*/*z* (%): 452 (M^+^, 46), 453 (M^+^ + 1, 38), 475 ([M + Na]^+^, 3). Elemental analysis: Calcd. for C_24_H_22_ClN_2_O_3_P: C, 63.65; H, 4.90; N, 6.19. Found: C, 63.65; H, 4.96; N, 6.22.

*Diphenyl N-(3-bromophenyl)amino(pyrrol-2-yl)methylphosphonate* (**2d**), Y = 0.275 g (57%), m.p. = 157–159 °C (white crystals). ^1^H NMR (600 MHz, DMSO-*d*_6_): δ 10.80 (s, NH_pyr_, 1H); 7.36–7.33 (m, H_Ph_, 2H); 7.31–7.28 (m, H_Ph_, 2H); 7.18 (t, ^3^*J*_HH_ = 7.3 Hz, H_Ph_, 1H); 7.15 (t, ^3^*J*_HH_ = 7.4 Hz, H_Ph_, 1H); 7.07–7.01 (m, H_Ph_, 4H); 6.85 (dd, ^4^*J*_HH_ = 2.3 and ^3^*J*_HH_ = 8.3 Hz, H_Ph_, 1H); 6.81 (d, ^3^*J*_HH_ = 8.0 Hz, H_Ph_, 2H); 6.76–6.74 (m, H_Ph_, H_pyr_, 3H); 6.63 (dd, ^4^*J*_HH_ = 3.1 and ^3^*J*_HH_ = 10.2 Hz, NH, 1H); 6.29–6.27 (m, H_pyr_, 1H); 6.01 (dd, ^3^*J*_HH_ = 2.8 and 5.7 Hz, H_pyr_, 1H); 5.53 (dd, ^2^*J*_PH_ = 22.8 and ^3^*J*_HH_ = 10.2 Hz, CHP, 1H). ^13^C NMR (150 MHz, DMSO-*d*_6_): δ 150.6 (d, ^2^*J*_PC_ = 10.5 Hz, POC); 150.5 (d, ^2^*J*_PC_ = 11.5 Hz, POC); 149.3 (d, ^3^*J*_PC_ = 12.0 Hz, PCNC); 131.1 (C_arom_); 130.24 (C^Ph^_meta_); 130.16 (C^Ph^_meta_); 125.7 (C^Ph^_para_); 125.6 (C^Ph^_para_); 124.7 (C^pyr^_ipso_); 122.7 (C_arom_); 121.9 C_Ph_); 121.1 (d, ^4^*J*_PC_ = 3.8 Hz, C^Ph^_ortho_); 120.9 (d, ^4^*J*_PC_ = 3.8 Hz, C^Ph^_ortho_); 120.2 (C_arom_); 118.6 (d, ^5^*J*_PC_ = 2.7 Hz, C^5^_pyr_); 116.3 (C_arom_); 113.0 (C_arom_); 108.7 (d, ^3^*J*_PC_ = 5.9 Hz, C^3^_pyr_); 108.4 (C^4^_pyr_); 49.2 (d, ^1^J_PC_ = 163.9 Hz, PC). ^31^P NMR (81 MHz, DMSO-*d*_6_): δ 15.08. IR (KBr): 3333, 3288 (νNH); 3132, 3061 (νCH_arom_); 1597, 1587, 1509, 1488, 1472, 1455 (νCC_arom_); 1239 (νP-O); 770 (∂CH_arom_); 688 (∂CC_arom_). ESI MS *m/z* (%): 482 (M^+^, 44), 483 (M^+^ + 1, 55), 505 ([M + Na]^+^, 99), 521 (([M + K]^+^, 75). Elemental analysis: Calcd. for C_23_H_20_BrN_2_O_3_P: C, 57.16; H, 4.17; N, 5.80. Found: C, 57.30; H, 4.24; N, 5.82.

*Diphenyl N-(1-naphthyl)amino(pyrrol-2-yl)methylphosphonate* (**2e**), Y = 0.291 g (64%), m.p. = 142–145 °C (white powder). ^1^H NMR (600 MHz, DMSO-*d*_6_): δ 11.05 (s, NH_pyr_, 1H); 8.18–8.17 (m, H_napht_, 1H); 7.80–7.79 (m, H_napht_, 1H); 7.49–7.46 (m, H_napht_, 2H); 7.31–7.25 (m, H_Ph_, H_napht_, 6H); 7.16 (t, ^3^*J*_HH_ = 7.4 Hz, H_Ph_, 1H); 7.15 (t, ^3^*J*_HH_ = 7.5 Hz, H_Ph_, 1H); 7.08–7.07 (m, H_Ph_, 2H); 6.93 (dd, ^4^*J*_HH_ = 1.5 and ^3^*J*_HH_ = 7.3 Hz, H_napht_, 1H); 6.82 (d, ^3^*J*_HH_ = 8.0 Hz, H_Ph_, 2H); 6.79–6.78 (m, H_pyr_, 1H); 6.40–6.38 (m, H_pyr_, 1H); 6.26 (dd, ^4^*J*_HH_ = 2.6 and ^3^*J*_HH_ = 10.7 Hz, NH, 1H); 6.02 (dd, ^3^*J*_HH_ = 2.8 and 5.6 Hz, H_pyr_, 1H); 5.75 (dd, ^2^*J*_PH_ = 23.9 and ^3^*J*_HH_ = 10.7 Hz, CHP, 1H). ^13^C NMR (150 MHz, DMSO-*d*_6_): δ 150.8 (d, ^2^*J*_PC_ = 10.0 Hz, POC); 150.7 (d, ^2^*J*_PC_ = 9.9 Hz, POC); 142.4 (d, ^3^*J*_PC_ = 13.0 Hz, PCNC); 134.4 (C_napht_); 130.20 (C^Ph^_meta_); 130.16 (C^Ph^_meta_); 128.5 (C_napht_); 126.8 (C_napht_); 126.3 (C_napht_); 125.61 (C^Ph^_para_); 125.55 (C^Ph^_para_); 125.0 (C_napht_); 124.7 (C_napht_); 124.5 (C^pyr^_ipso_); 122.2 (C_napht_); 121.0 (d, ^4^*J*_PC_ = 3.8 Hz, C^Ph^_ortho_); 120.9 (d, ^4^*J*_PC_ = 3.8 Hz, C^Ph^_ortho_); 118.9 (d, ^5^*J*_PC_ = 2.8 Hz, C^5^_pyr_); 118.6 (C_napth_); 109.2 (d, ^3^*J*_PC_ = 7.4 Hz, C^3^_pyr_); 108.2 (C^4^_pyr_); 107.0 (C_napht_); 50.3 (d, ^1^*J*_PC_ = 163.9 Hz, PC). ^31^P NMR (243 MHz, DMSO-*d*_6_): δ 15.14. IR (KBr): 3345, 3270 (νNH); 3057 (νCH_arom_); 1589, 1524, 1489, 1455 (νCC_arom_); 1239 (νP-O); 766 (∂CC_arom_). ESI MS *m*/*z* (%): 454 (M^+^, 49), 455 (M^+^ + 1, 31), 477 ([M + Na]^+^, 8), 477 (([M + K]^+^, 5). Elemental analysis: Calcd. for C_27_H_23_N_2_O_3_P: C, 71.36; H, 5.10; N, 6.16. Found: C, 71.16; H, 5.13; N, 6.20.

### 4.2. Evaluation of Ecotoxicity of Compounds ***2a**–**e***

#### 4.2.1. Microtox^®^ Toxicity Assay

The detailed procedure of the Microtox toxicity assay has been described previously by Lewkowski et al. [45]. The method is based on the analysis of light emission reduction of luminescent bacteria (*Aliivibrio fischeri*) under toxic stress. The tests were carried out in a Microtox^®^ M500 analyzer according to the 1992 Microtox^®^ manual. The Microtox^®^ solid-phase test (MSPT) was adopted in the report of Doe et al. [39].

The MSPT procedure allows the test organisms to come into direct contact with the solid sample in an aqueous suspension of the test sample. Thus it is possible to detect toxicity which is due to the insoluble solids that are not in the solution. All materials and reagents were purchased from MODERNWATER (New Castle, DE, USA). Toxicity was determined by using the marine luminescent bacterium, *Aliivibrio fischeri*, naturally adapted to a saline environment. Briefly, bacteria were regenerated with 1 mL of reconstitution solution (0.01%) and placed in the reagent well of the Microtox. A suspension of 7 g of the sediment was prepared in 35 mL of a solid phase diluent (3.5% NaCl) and was magnetically stirred for 10 min. Then a series of dilutions were made and bacteria (approx. 1 × 10^6^ cell/mL per assay) were exposed to these dilutions and to a blank (3.5% NaCl solution) for 20 min. Next, after filtration, the light output of supernatants containing exposed bacteria was measured after 5 min with a Microtox^®^ Analyzer 500. Inhibition was calculated as the concentration of the compound loaded to sediment (mg/L) that caused a 50% reduction in the light emitted by the bacteria, and EC_50_ along with 95% confidence limit determined by the software provided with the analyzer.

#### 4.2.2. Ostracod Test Kit

Ecotoxicity evaluation of the synthesized compounds was performed in a short-term contact test using Ostracodtoxkit F^TM^ provided by MicroBiotests Inc., Belgium. This direct sediment contact bioassay was performed in multiwell test plates using neonates of the benthic ostracod crustacean *Heterocypris incongruens* hatched from cysts [46]. After six days in contact with the sediment (or soil) the percentage mortality and the growth of the crustaceans were determined and compared to the results obtained in a non-treated reference sediment (soil).

Briefly, according to the manual of the Ostracodtoxkit test, the cysts (*Heterocypris incongruens*) were transferred into a Petri dish filled with 10 mL standard fresh water (reconstituted water) and were incubated at 25 °C for 52 h under continuous illumination (approx. 3000–4000 lux).

After 48 h of cyst incubation, pre-feeding of the freshly-hatched ostracods was performed with algae (spirulina-powder) provided in the test kit. Next, after hatching, before feeding with algal food suspension, the length measurements of ostracod neonates was conducted. Algae (*Selenastrum capricornutum*) used as feed in the test plate were reconstituted according to the manufacturer’s procedure. Each well of a test plate was filled in the following order: 2 mL standard freshwater, 2 × 500 μL of sediment (soil) treated and non-treated for comparison (blank), 2 mL already-prepared algal suspension, 10 ostracods. The test plates were covered with Parafilm^®^ and closed with a lid. Then, multiwall plates were incubated at 25 °C in darkness for six days. After six days of exposure, the ostracods were recovered from the multiwells to determine the percentage mortality. To calculate the growth inhibition of survived organisms, their length measurements were also conducted. The mortality of test organisms was determined in six replicates. The measurement of length was carried out by means of a micrometric strip placed on the bottom of a glass microscope plate. Growth inhibition (GI) of *H. incongruens* in the test sediment was calculated as follows:

$$\%\;growth\;inhibition=100-\frac{growth\;in\;test\;sed.}{growth\;in\;ref.sed.}\times 100\%$$

Statistical differences between variables were analyzed with ANOVA.

## 5. Conclusions

To conclude, we have synthesized five new pyrrole-derived, diphenyl aminophosphonates and we preliminarily evaluated their impact on the environment by testing the action on *A. fischeri* bacteria and crustaceans *H. incongruens*. All investigated compounds exhibited ecotoxicity against tested organisms, but to a different degree. Moreover, a certain pattern was observed. Those compounds which were found to be strongly toxic for *H. incongruens* showed rather slight toxicity against *A. fischeri*, and vice-versa. Interestingly, a structure vs. activity pattern has also been noticed, i.e., when the *N*-substituent was an unsubstituted aryl group (1-naphthyl, phenyl), compounds **2a** and **2e** were significantly toxic for ostracods, being simultaneously almost harmless for bacteria. On the other hand, an *N*-(4-nitrophenyl) derivative **2b** was relatively safe for crustaceans, but it was highly toxic for *A. fischeri*. The latter could be expected, because the impact of a nitro group on bacteria is quite well known, but it is characteristic for a nitro group linked to imidazolyl or furyl moieties. Compounds **2c**–**d**, which do not belong to any of mentioned groups, were equally toxic for both tested organisms, whereby their toxicity may be considered as moderate.

Studies on these compounds will be deepened in the near future and extended on, e.g., the investigation of their binding to some plant proteins or in the aspect of their prospective pharmacological applications. However, these data clearly demonstrate that any possible application of studied compounds **2a**–**b** and **2e** (or any other compound similar to them) require the special care and results described by the approach in this paper to the knowledge of how to handle their wastes.

## Figures and Tables

**Table 1:** Microtox^®^ EC_50_ values (mg/kg of soil dry weight) of exposure of the luminescent marine bacteria *Aliivibrio fischeri* to tested aminophosphonates **2a**–**e** with, respectively, 95% confidence limits (in brackets) obtained in the fit of the data.

Compounds	EC_50_ (Lower Limit; Upper Limit)	Coefficient of Determination (R^2^)
**2a**	2262 (1967; 2602)	0.9715
**2b**	750.9 (685.6; 810.5)	0.9918
**2c**	1656 (1465; 1871)	0.9785
**2d**	1141 (846.5; 1537)	0.8998
**2e**	5365 (4232; 6801)	0.9796

**Table 2:** Growth inhibition (%) of *Heterocypris incongruens* living in soil contaminated with aminophosphonates **2a**–**e** at concentrations of 10, 50, 100, and 250 mg/kg of soil dry weight. SD means standard deviation (SD, *n* = 6 determinations). NM means not measured because mortality was higher than 30%.

Concentration of the Compound [mg/kg of Soil d.w.]	Growth Inhibition [%]				
	**2a**	**2b**	**2c**	**2d**	**2e**
Control	0	0	0	0	0
10	16 ± 2	2 ± 1	3 ± 1	2 ± 1	19 ± 2
50	NM	21 ± 2	31 ± 2	26 ± 3	NM
100	NM	NM	NM	NM	NM
250	NM	NM	NM	NM	NM

## Supplementary Materials

Supplementary Materials are available online, Figures S1–S5: Sets of NMR spectra of compounds **2a**–**e**; Figure S6: FT-IR spectra of aminophosphonates **2a**–**e**; Figure S7: ESI-MS spectra of aminophosphonates **2a**–**e**.
