# Supplementary material for: Novel N-Arylaminophosphonates Bearing a Pyrrole Moiety and Their Ecotoxicological Properties
Source: Molecules. 2017 Jul 7;22(7):1132. doi: 10.3390/molecules22071132 (PMC6152063; doi:10.3390/molecules22071132)
Supplement: Supplementary file 1 [file molecules-22-01132-s001.pdf]

Novel, N-arylaminophosphonates Bearing a Pyrrole Moiety and Their Ecotoxicological Properties

Jarosław Lewkowski [1,*], Marta Morawska [1], Anna Kaczmarek [1,3], Diana Rogacz [2] and Piotr Rychter [2,*]

[1] Department of Organic Chemistry, Faculty of Chemistry, University of Łódź, Tamka 12, 91-403 Łódź, Poland; mz.morawska@gmail.com (M.M.)

[2] Faculty of Mathematics and Natural Science, Jan Długosz University in Częstochowa, 13/15 Armii Krajowej Av., 42-200 Częstochowa, Poland; p.rychter@ajd.czest.pl (P.R.); diana.rogacz@gmail.com (D.R.)

[3] M.Sc. student at the Faculty

* Correspondence: jlewkow@uni.lodz.pl; Tel.: +48-42-635-5751

Contents

Figure S1. ^{1}H NMR (**a**), COSY (**b**), ^{13}C NMR (**c**), HMQC (**d**) and ^{31}P NMR (**e**) spectra of diphenyl *N*-phenylamino(pyrrol-2-yl)-methylphosphonate (**2a**).

Figure S2. ^{1}H NMR (**a**), ^{13}C NMR (**b**) and ^{31}P NMR (**c**) spectra of diphenyl *N*-(4-nitrophenyl)-amino(pyrrol-2-yl)methylphosphonate (**2b**).

Figure S3. ^{1}H NMR (**a**), ^{13}C NMR (**b**) and ^{31}P NMR (**c**) spectra of diphenyl *N*-(4-chloro-2-methylphenyl)amino-(pyrrol-2-yl)methylphosphonate (**2c**).

Figure S4. ^{1}H NMR (**a**), ^{13}C NMR (**b**) and ^{31}P NMR (**c**) spectra of diphenyl *N*-(3-bromophenyl)-amino(pyrrol-2-yl)methylphosphonate (**2d**).

Figure S5. ^{1}H NMR (**a**), ^{13}C NMR (**b**) and ^{31}P NMR (**c**) spectra of diphenyl *N*-(1-naphthyl)amino-(pyrrol-2-yl)methylphosphonate (**2e**).

Figure S6 a–e. FT-IR spectra of aminophosphonates **2a–e**

Figure S7 a–e. ESI-MS spectra of aminophosphonates **2a–e**.

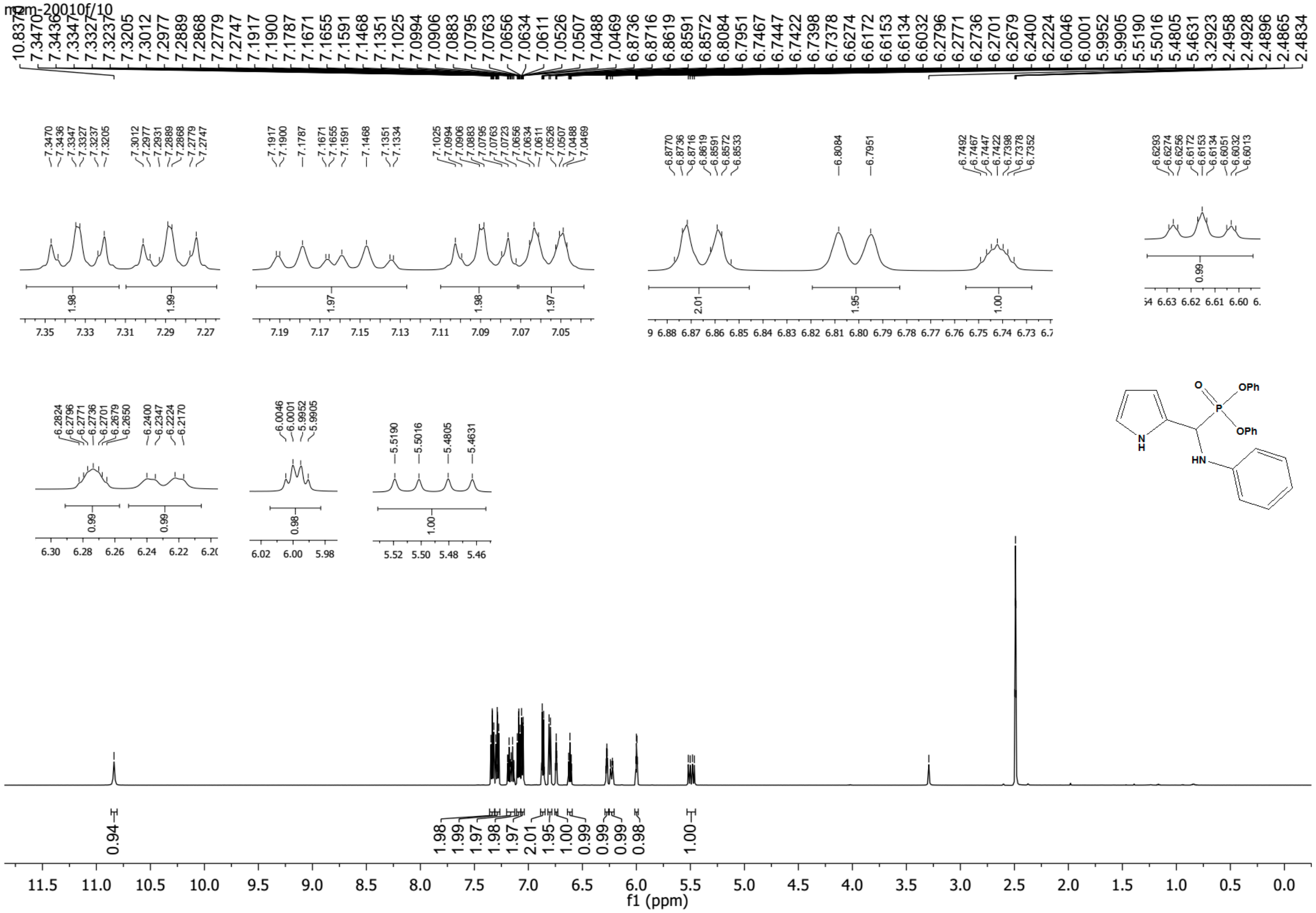

Figure S1. 1H NMR spectrum of diphenyl N-phenylamino(pyrrol-2-yl)-methylphosphonates (**2a**)

Figure S1a. ^{1}H-^{1}H COSY spectrum of diphenyl *N*-phenylamino(pyrrol-2-yl)-methylphosphonate (2a)- general review

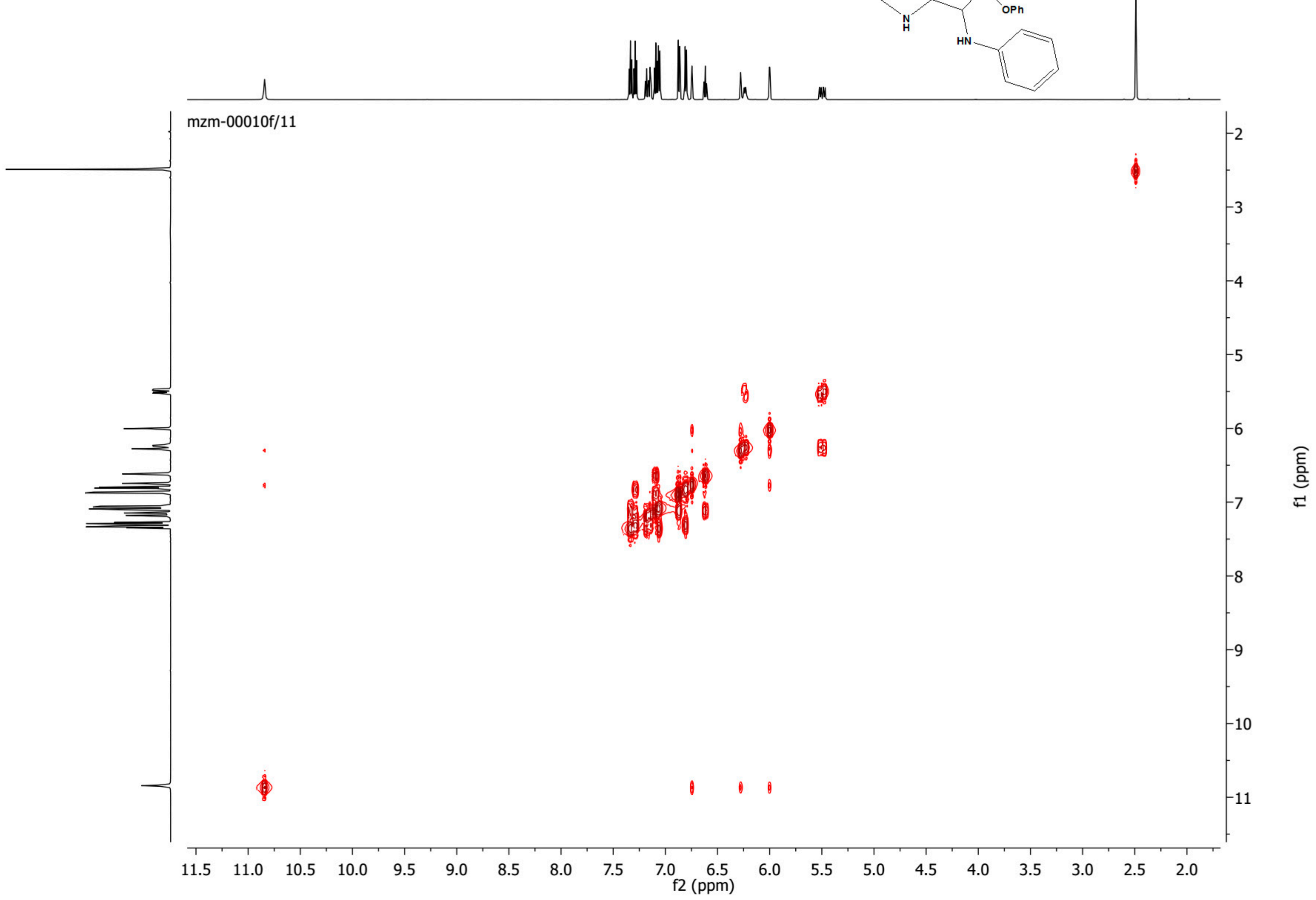

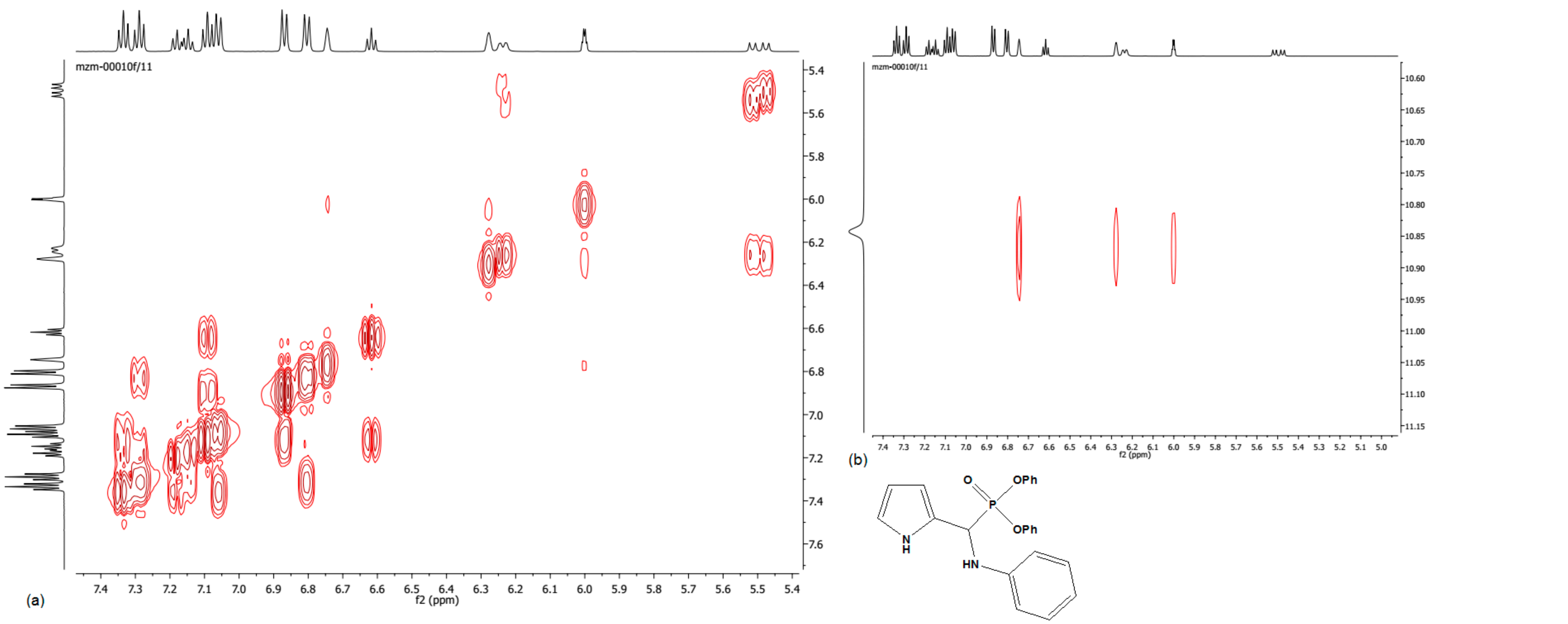

Figure S1b (bis). ^{1}H-^{1}H COSY spectrum of diphenyl *N*-phenylamino(pyrrol-2-yl)-methylphosphonate (**2a**): (a) 7.5 – 5.4 ppm region and (b) correlations in pyrrole ring

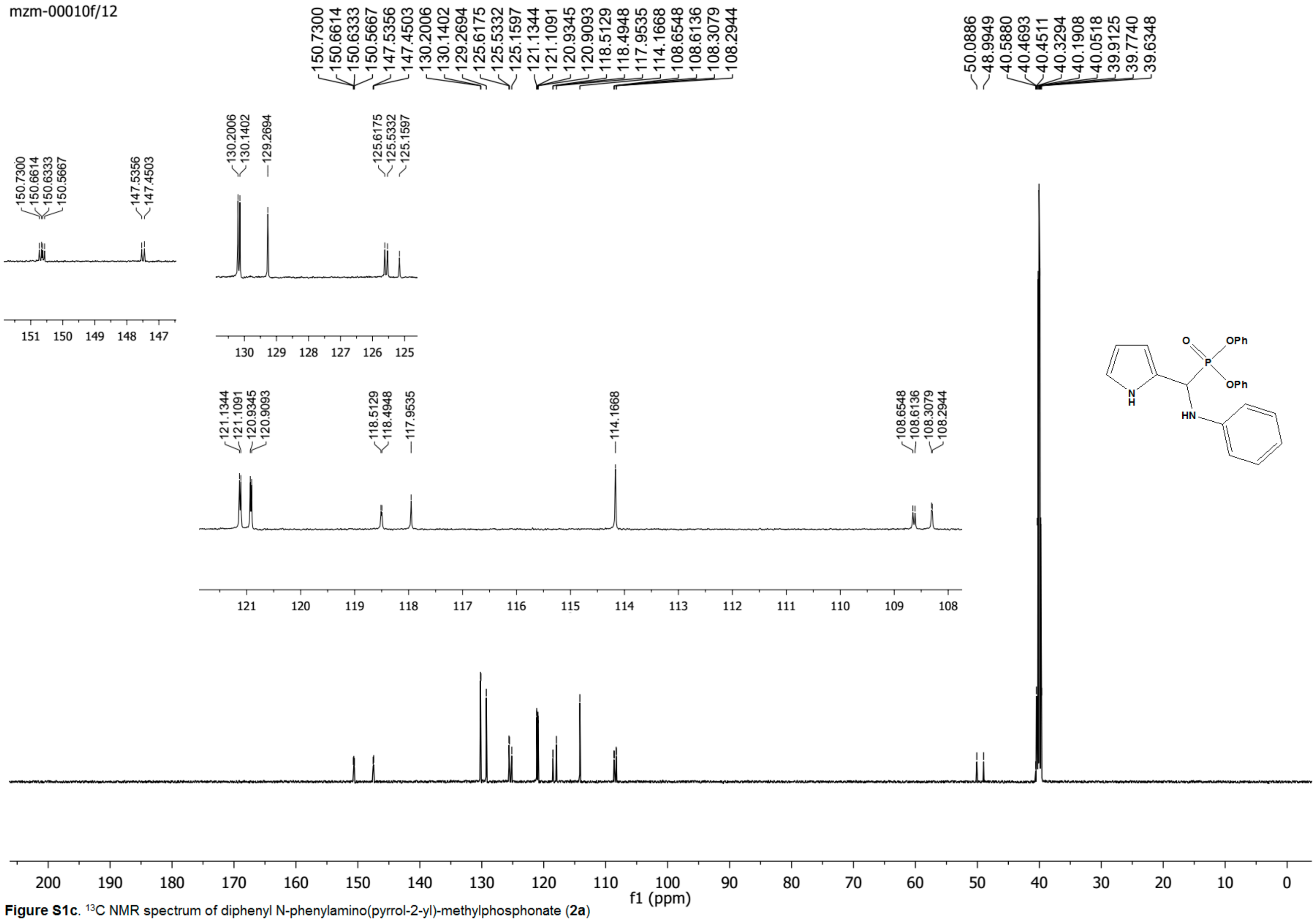

Figure S1c. ^{13}C NMR spectrum of diphenyl N-phenylamino(pyrrol-2-yl)-methylphosphonate (**2a**)

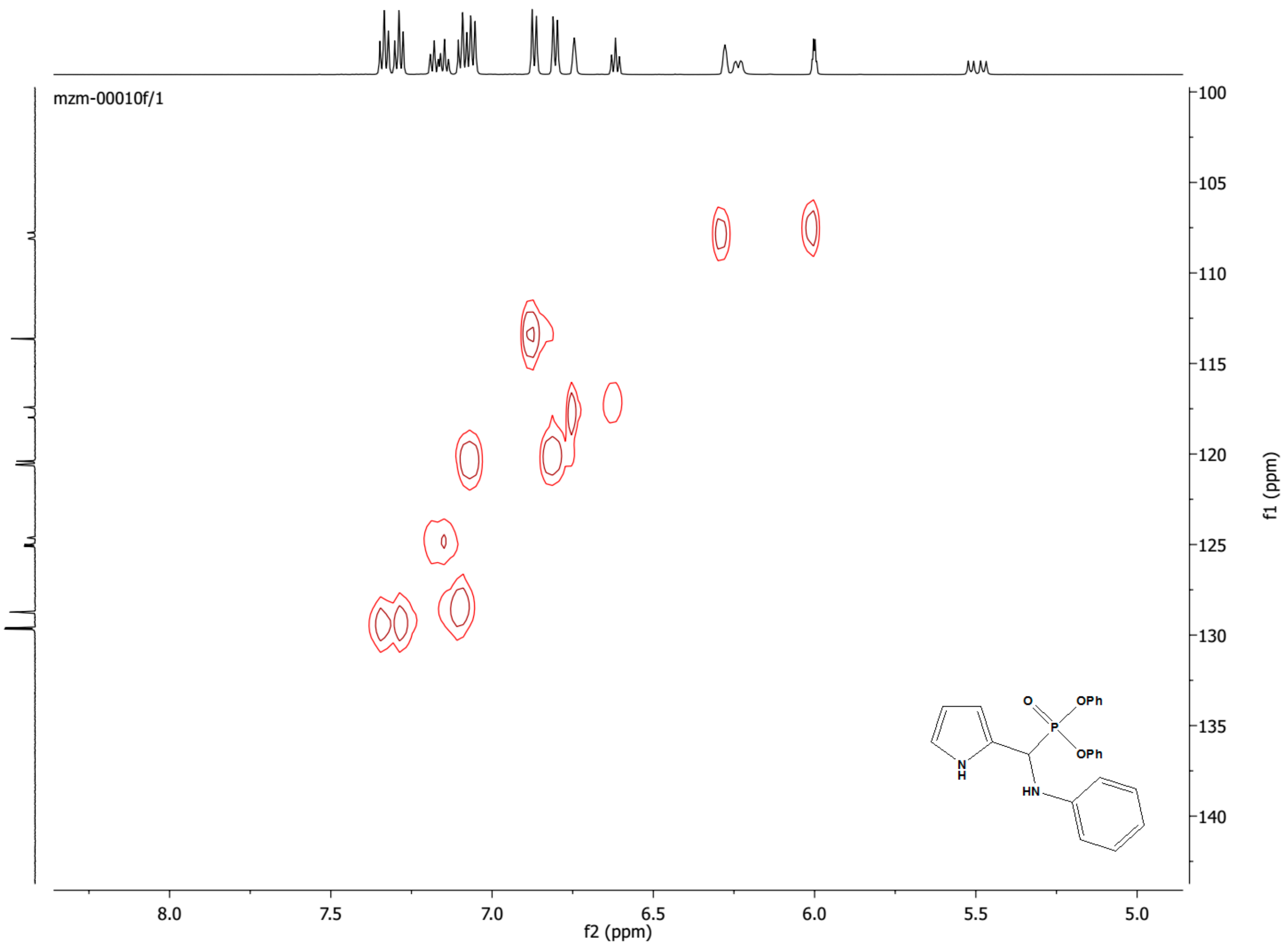

Figure S1d. ^{1}H-^{13}C HMQC spectrum of diphenyl N-phenylamino(pyrrol-2-yl)-methylphosphonate (**2a**)

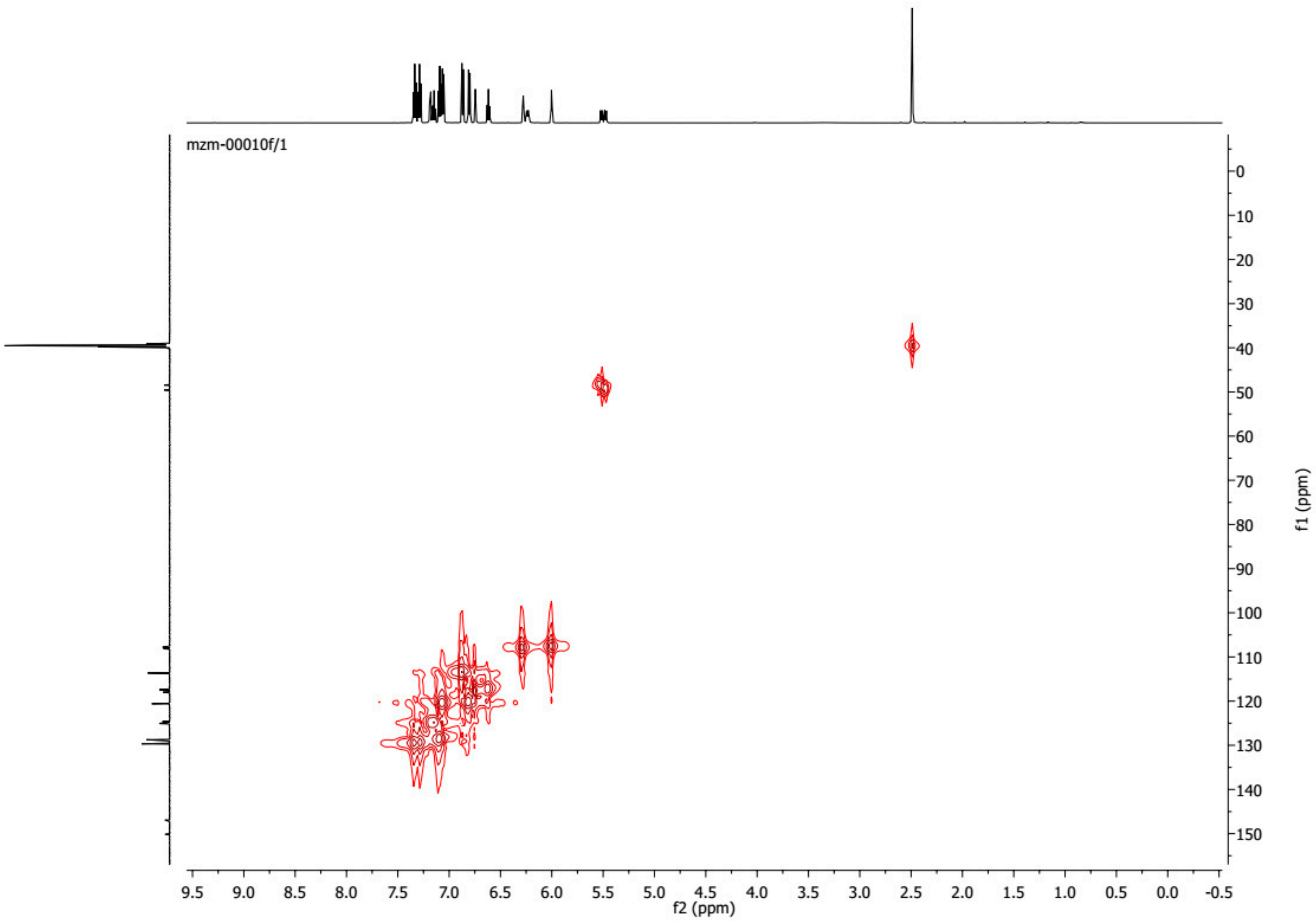

Figure S1d ***cont.***

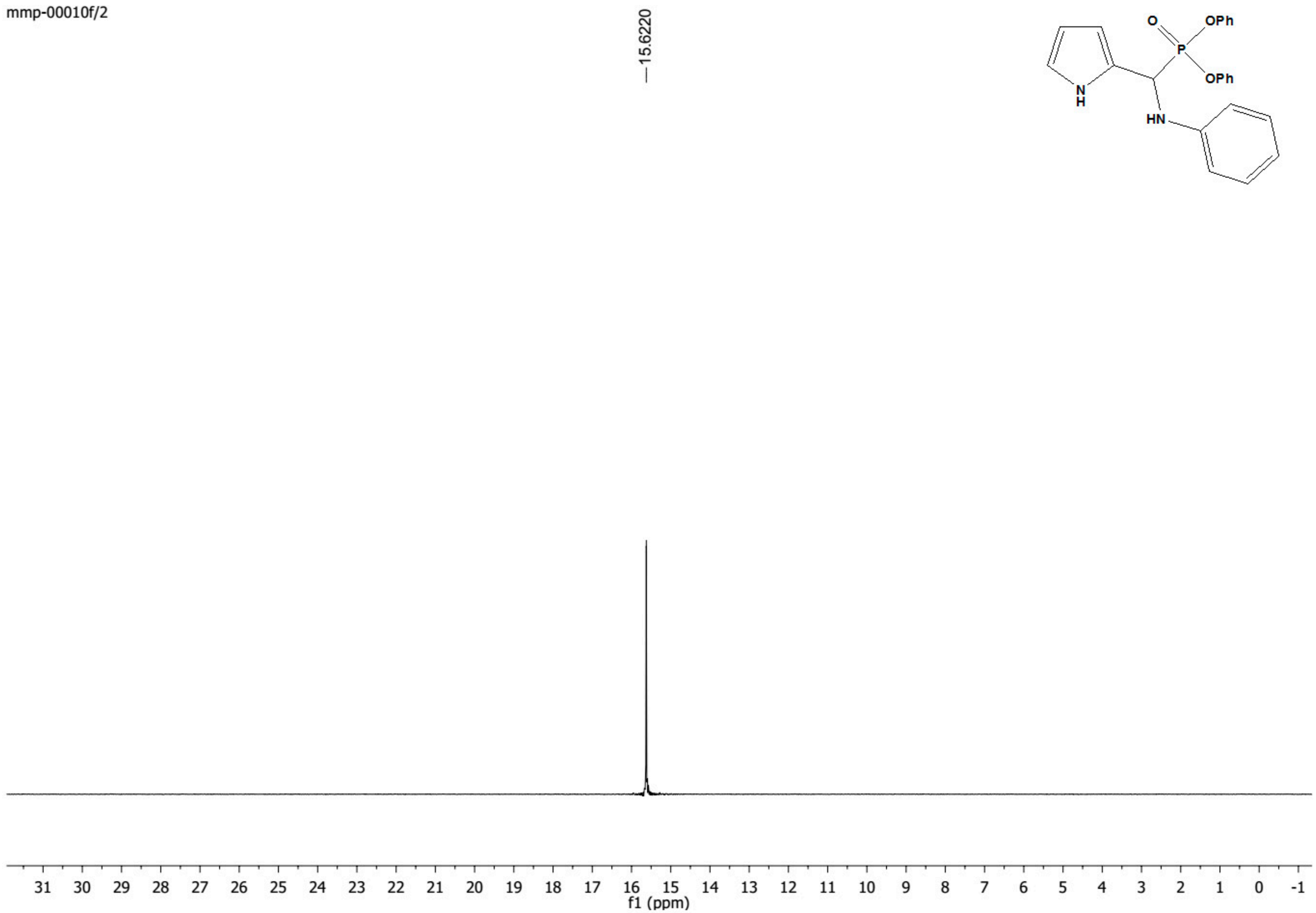

Figure S1e. ^{31}P NMR spectrum of diphenyl N-phenylamino(pyrrol-2-yl)-methylphosphonate (**2a**)

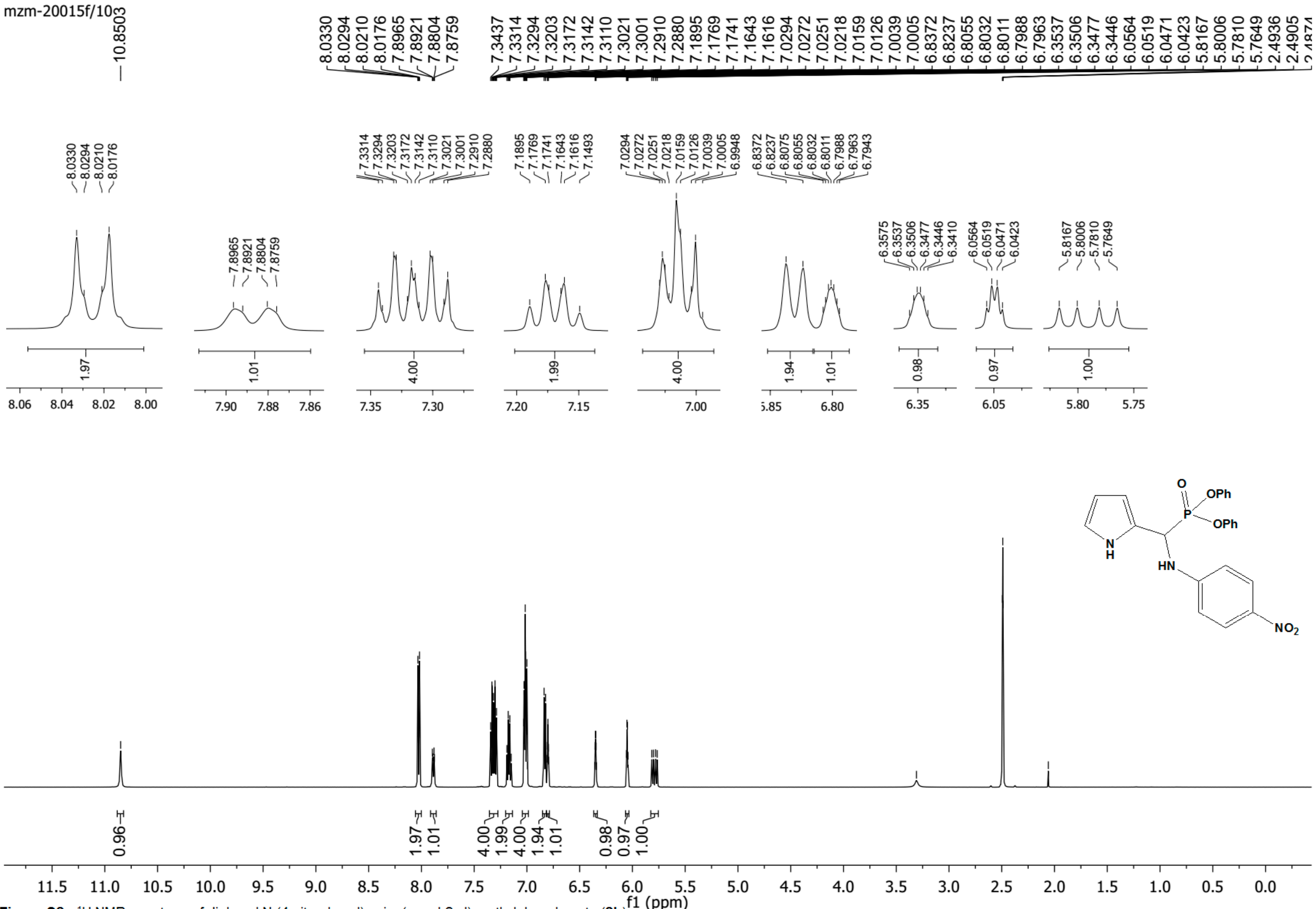

Figure S2a. ^1H NMR spectrum of diphenyl N-(4-nitrophenyl)amino(pyrrol-2-yl)-methylphosphonate (**2b**)

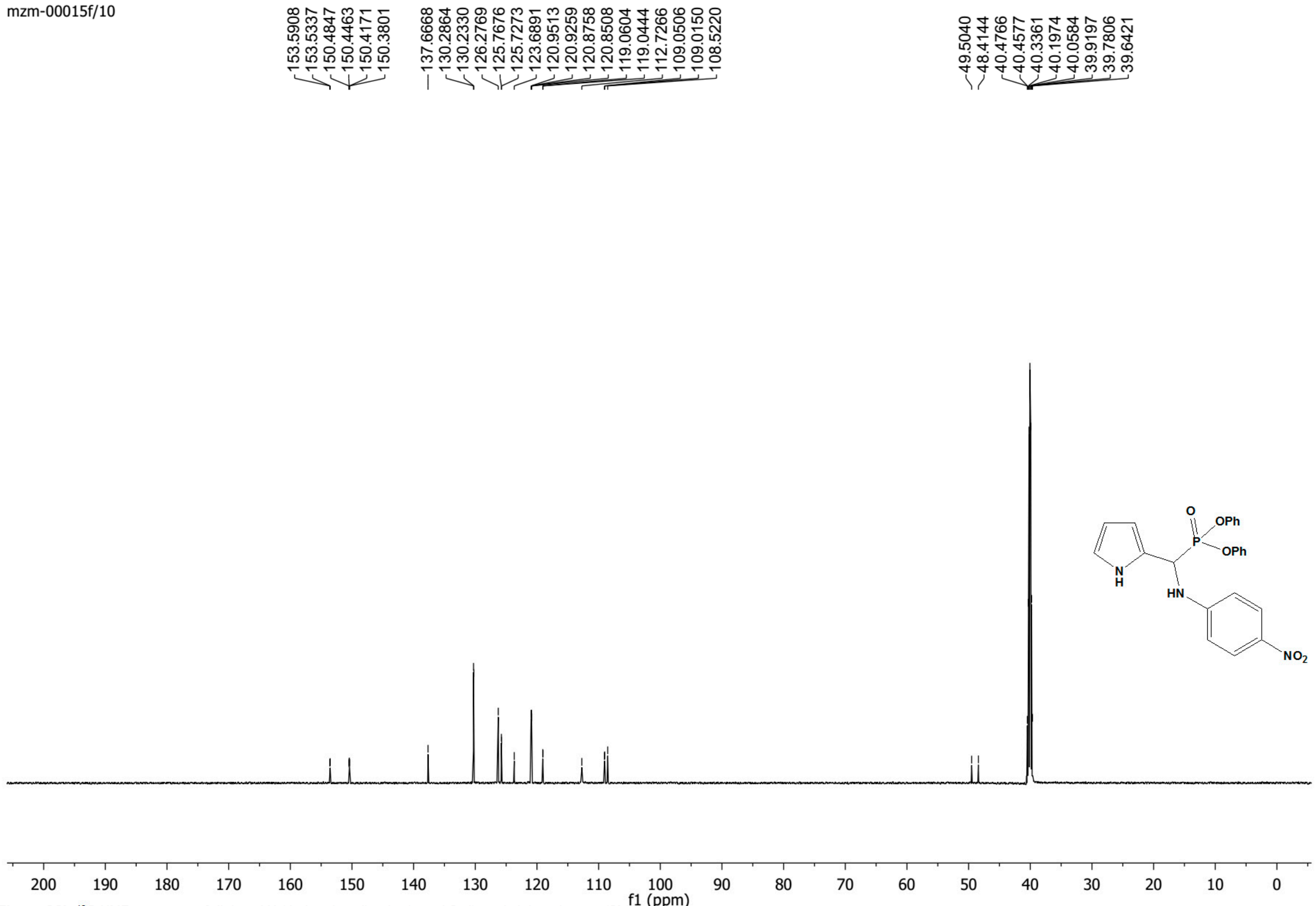

Figure S2b ^{13}C NMR spectrum of diphenyl N-(4-nitrophenyl)amino(pyrrol-2-yl)-methylphosphonate (**2b**)

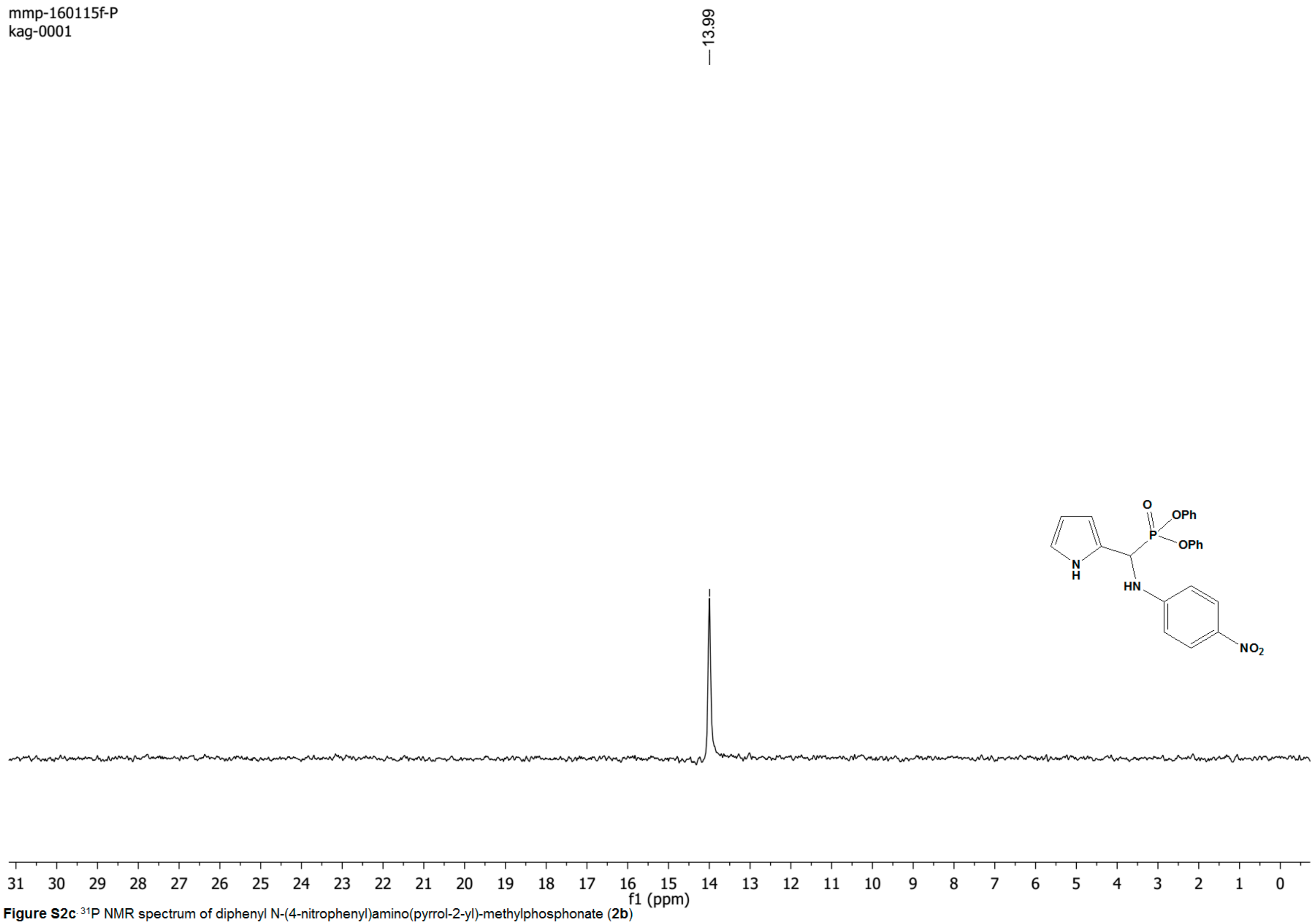

Figure S2c ^{31}P NMR spectrum of diphenyl N-(4-nitrophenyl)amino(pyrrol-2-yl)-methylphosphonate (**2b**)

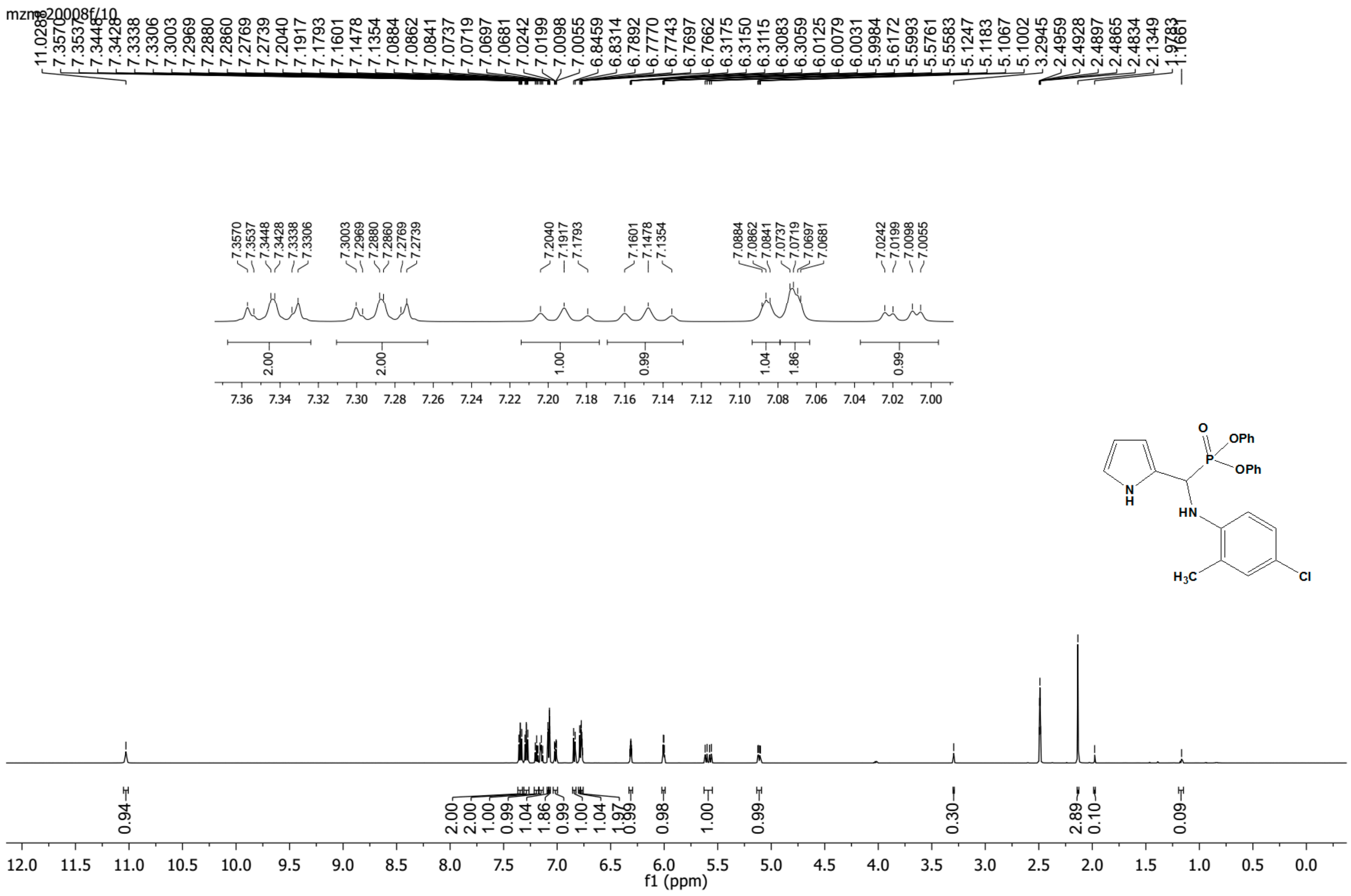

Figure S3a ^{1}H NMR spectrum of diphenyl N-(4-chloro-2-methylphenyl)amino(pyrrol-2-yl)-methylphosphonate (**2c**)

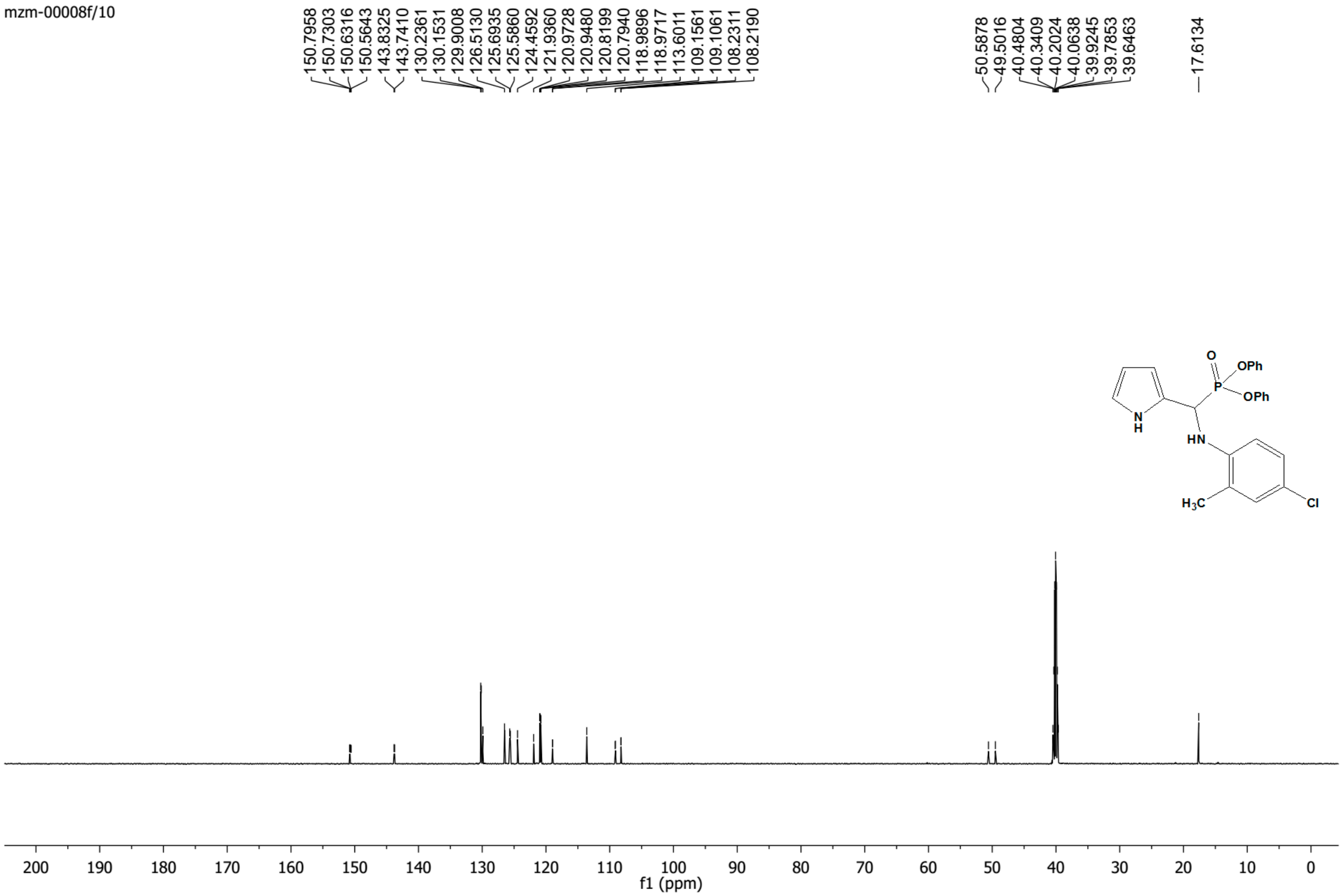

Figure S3b. ^{13}C NMR spectrum of diphenyl N-(4-chloro-2-methylphenyl)amino(pyrrol-2-yl)-methylphosphonate (**2c**)

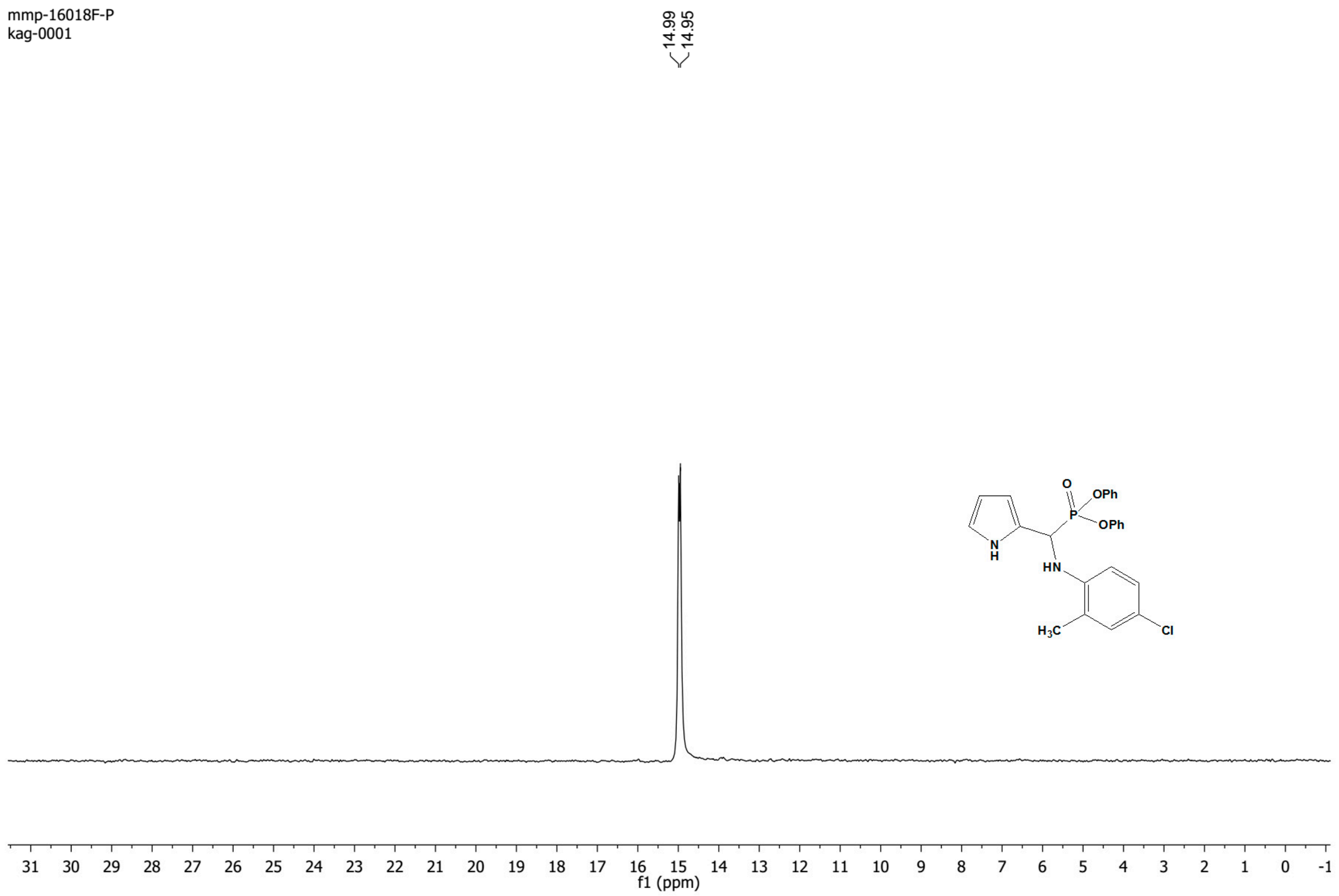

Figure S3c ^{31}P NMR spectrum of diphenyl N-(4-chloro-2-methylphenyl)amino(pyrrol-2-yl)-methylphosphonate (**2c**)

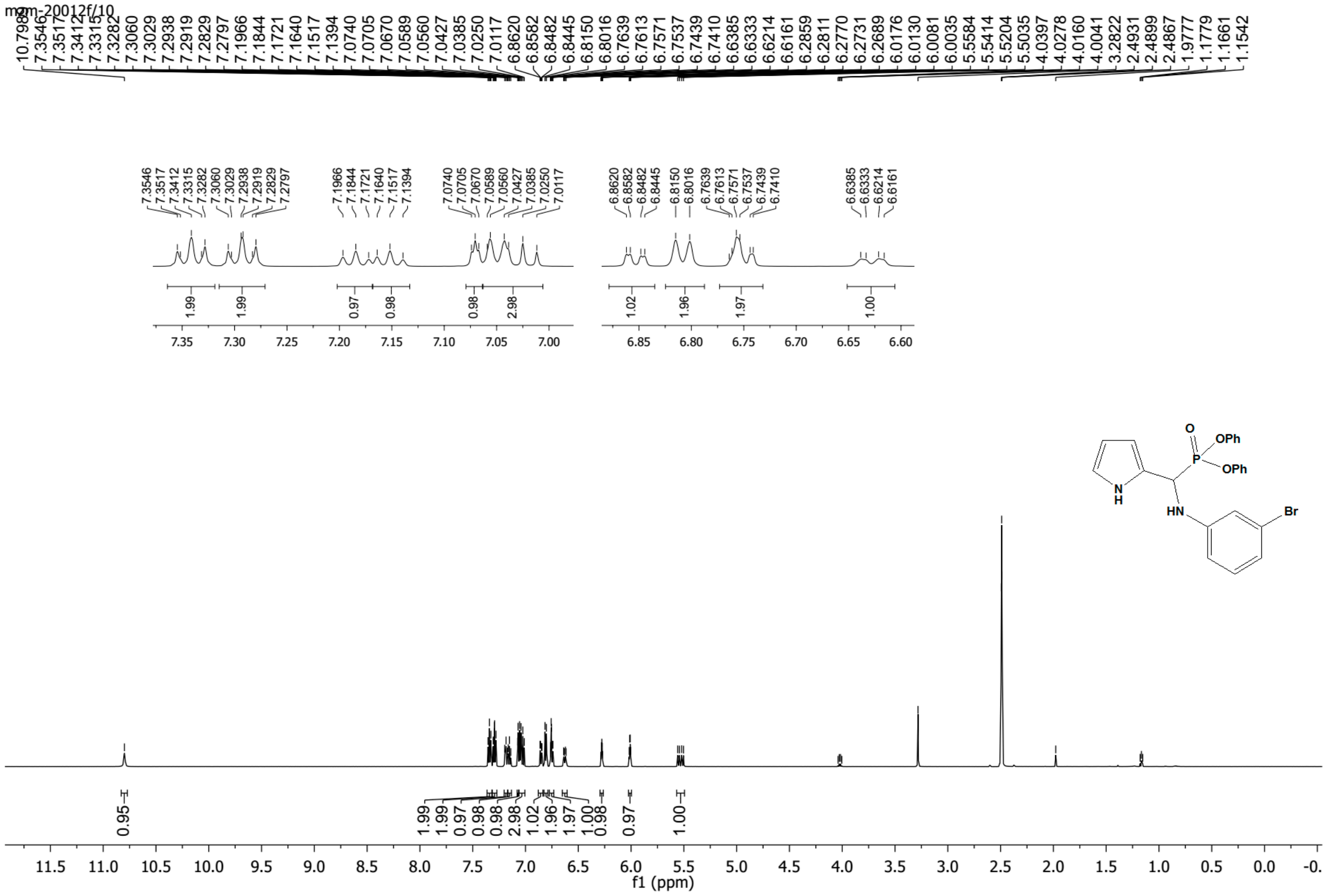

Figure S4a. ^{1}H NMR spectrum of diphenyl N-(3-bromophenyl)amino(pyrrol-2-yl)-methylphosphonate (**2d**)

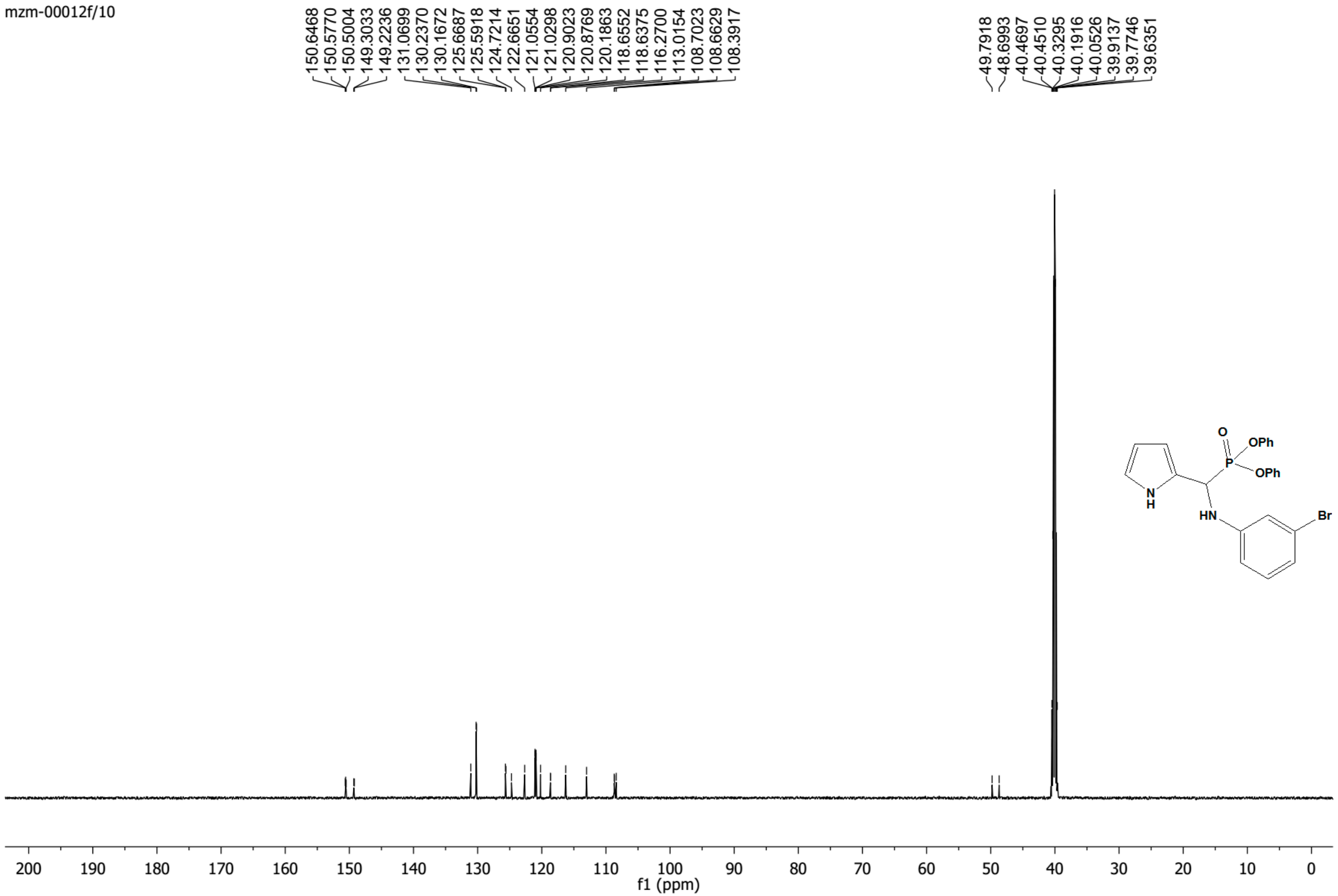

Figure S4b ^{13}C NMR spectrum of diphenyl N-(3-bromophenyl)amino(pyrrol-2-yl)-methylphosphonate (**2d**)

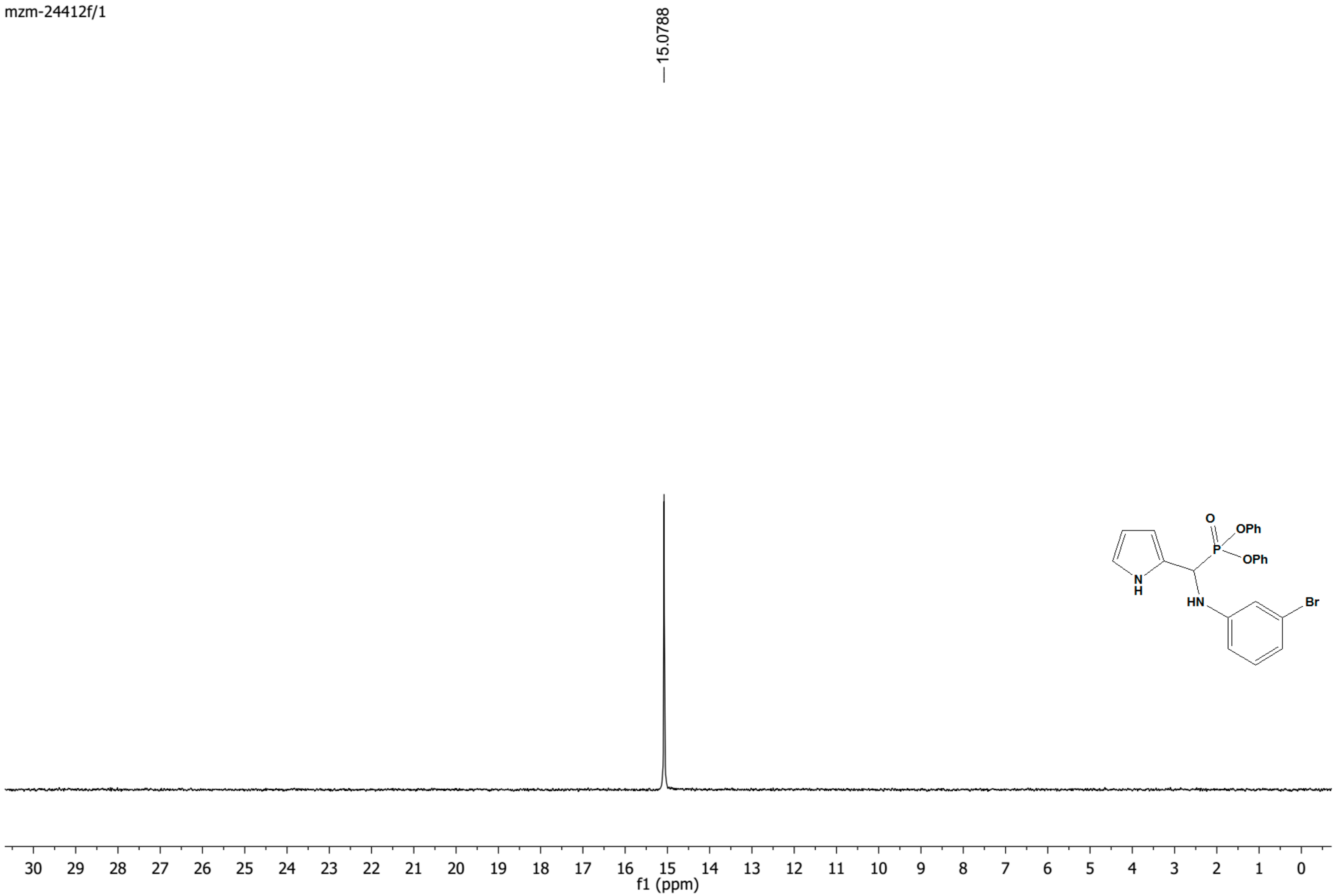

Figure S4c ^{31}P NMR spectrum of diphenyl N-(3-bromophenyl)amino(pyrrol-2-yl)-methylphosphonate (**2d**)

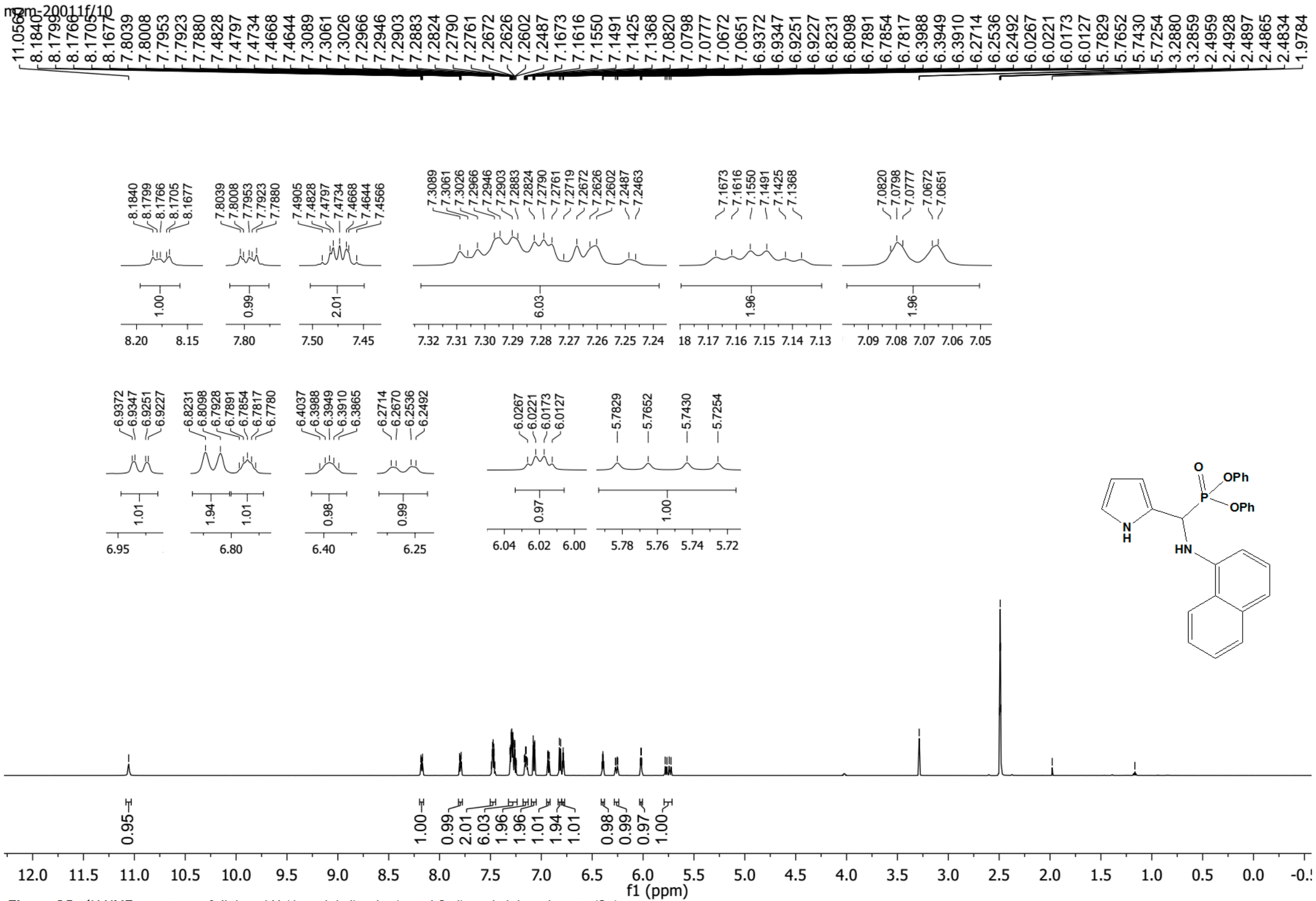

Figure S5a ^{1}H NMR spectrum of diphenyl N-(1-naphthyl)amino(pyrrol-2-yl)-methylphosphonate (**2e**)

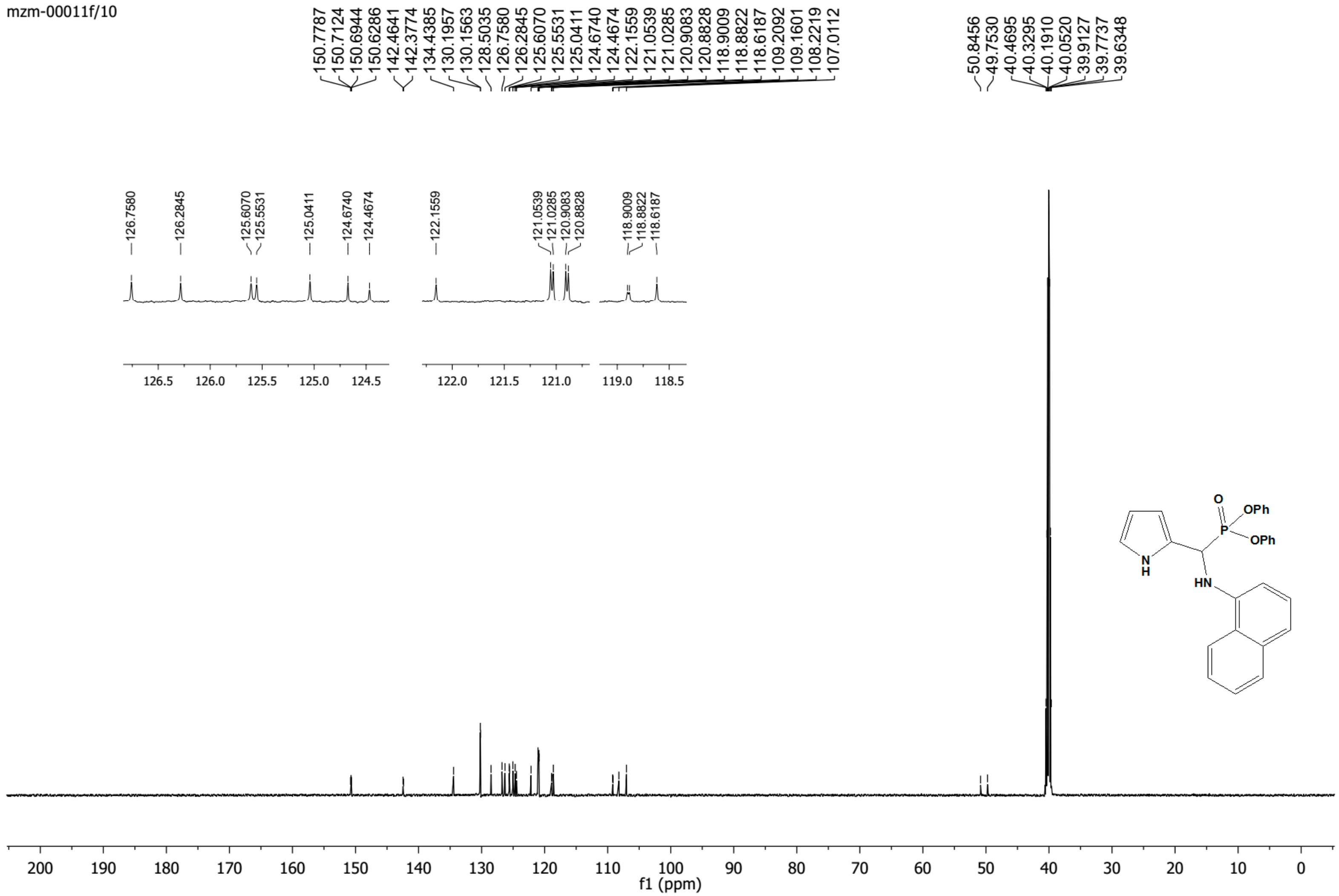

Figure S5b. ^{13}C NMR spectrum of diphenyl N-(1-naphthyl)amino(pyrrol-2-yl)-methylphosphonate (**2e**)

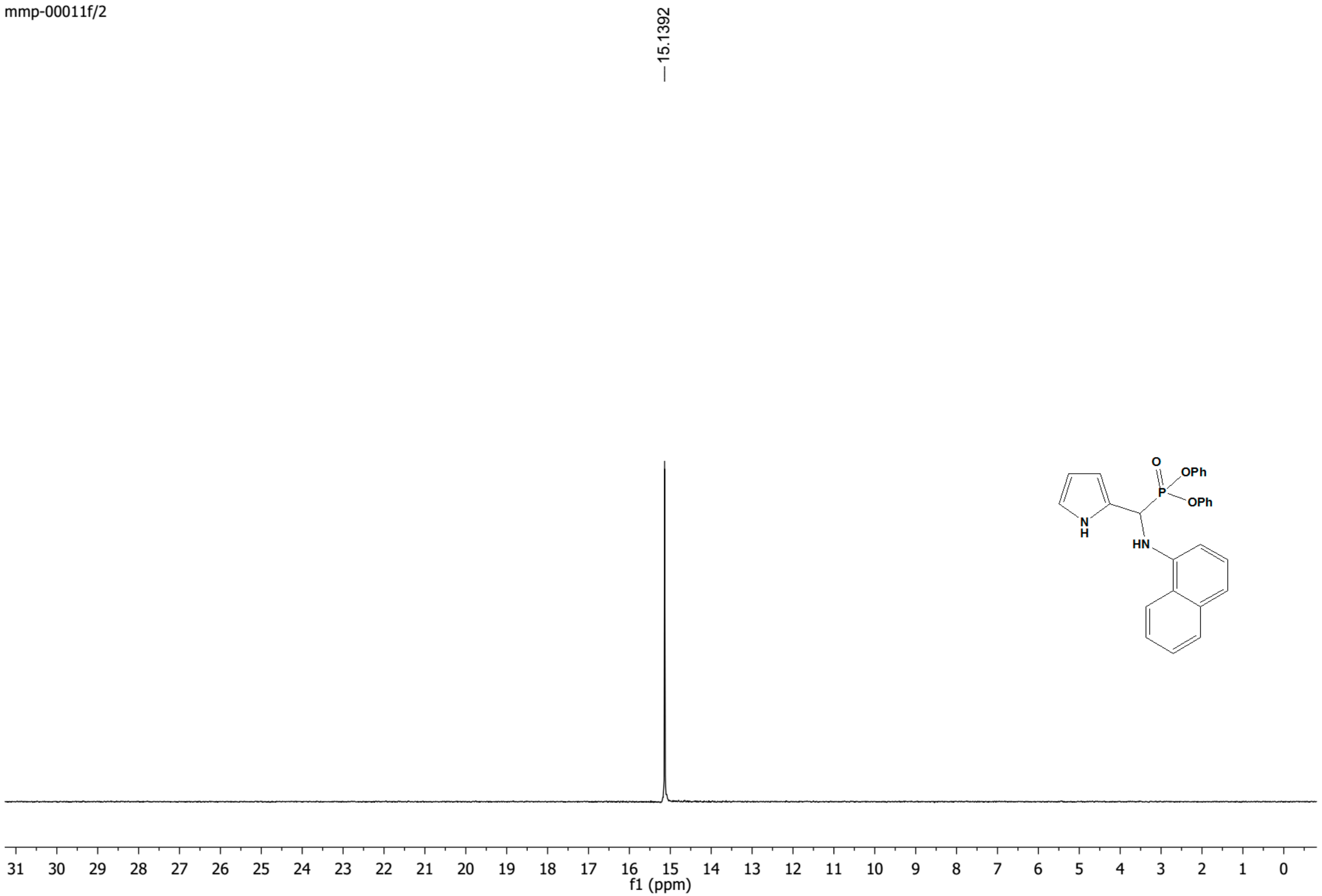

Figure S5c ^{31}P NMR spectrum of diphenyl N-(1-naphthyl)amino(pyrrol-2-yl)-methylphosphonate (**2e**)

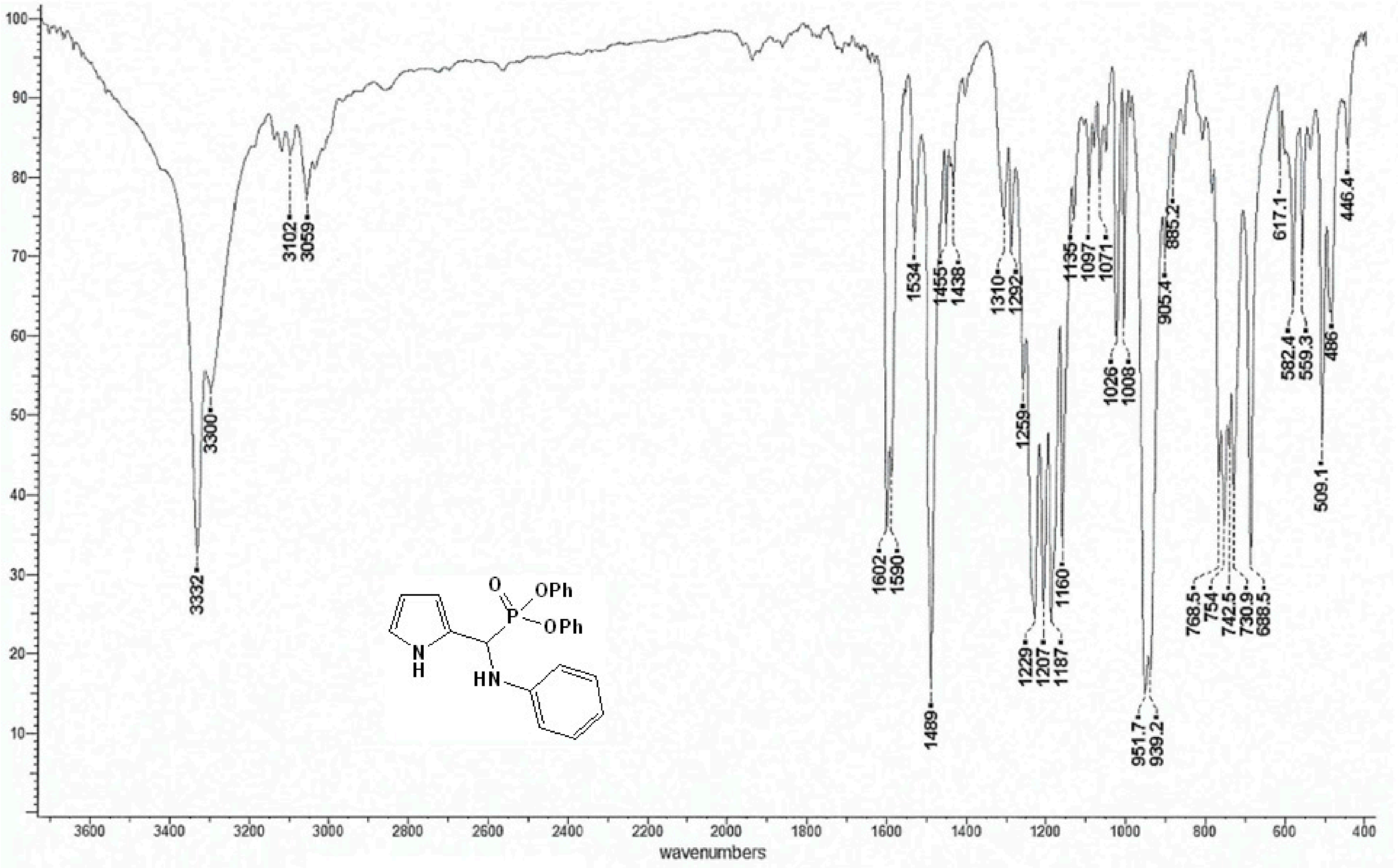

Figure S6a. FT-IR spectra of diphenyl *N*-phenylamino(pyrrol-2-yl)-methylphosphonate **2a**

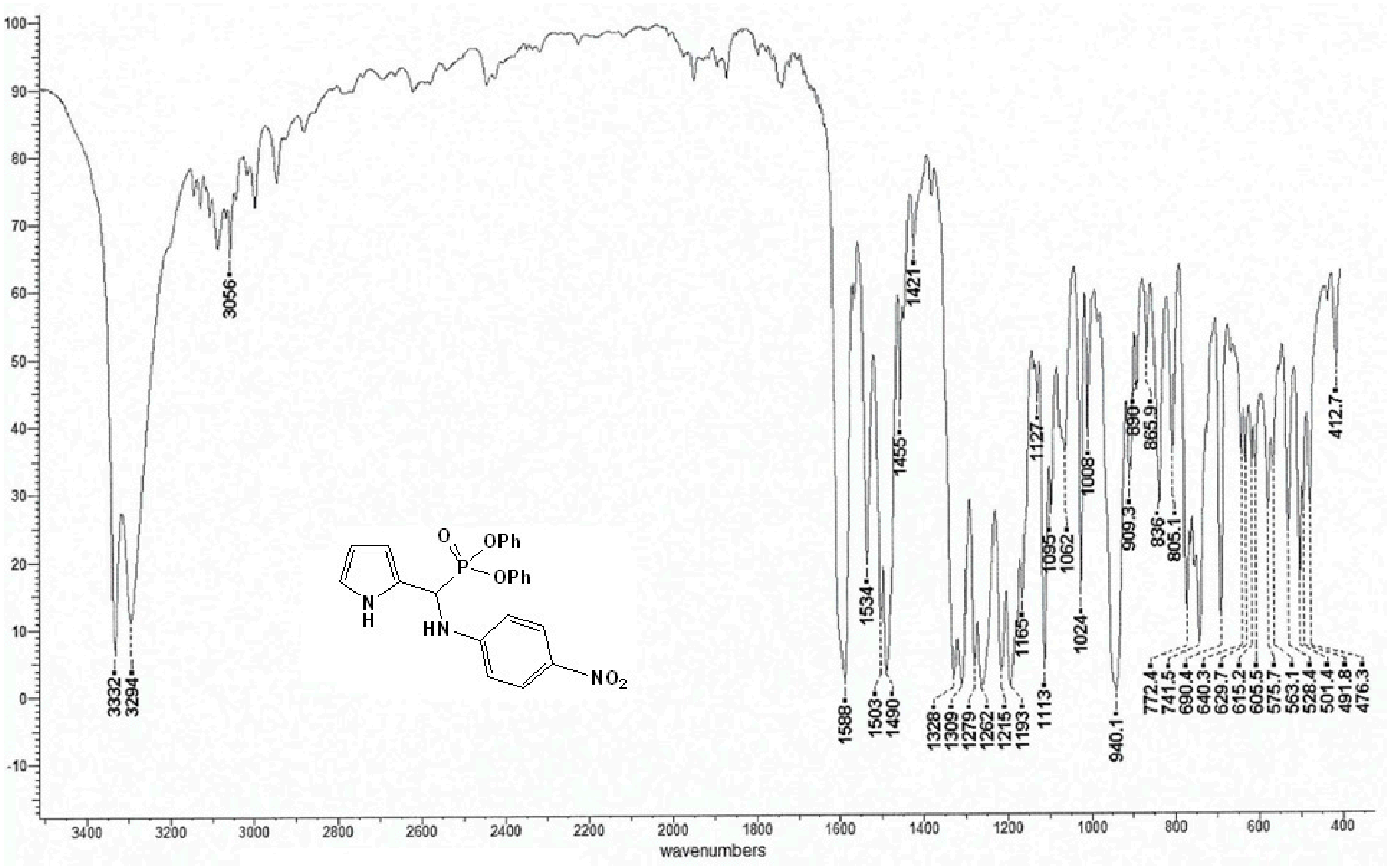

Figure S6b. FT-IR spectra of diphenyl N-(4-nitrophenyl)-amino(pyrrol-2-yl)methylphosphonate **2b**

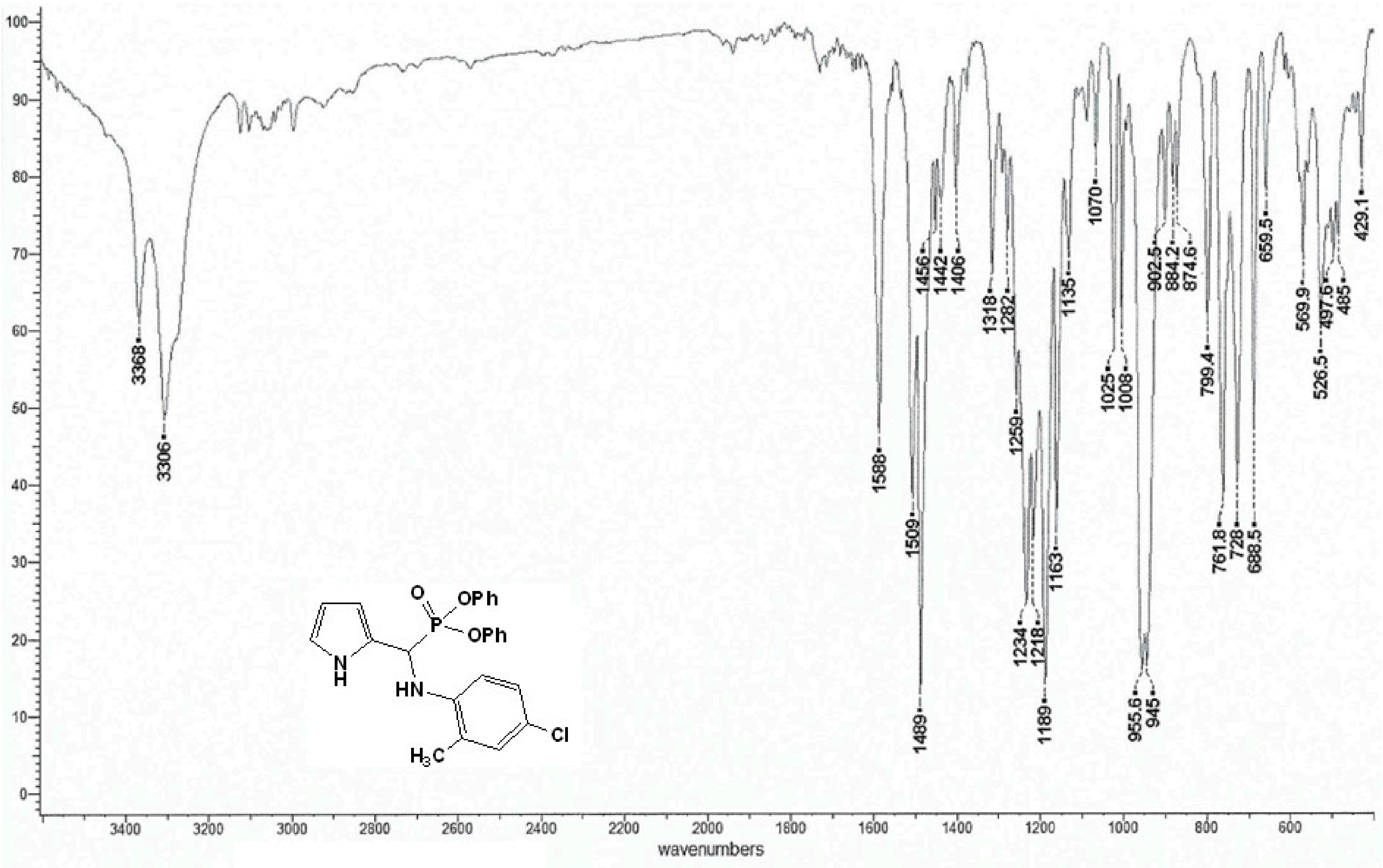

Figure S6c. FT-IR spectra of diphenyl N-(4-chloro-2-methylphenyl)amino-(pyrrol-2-yl)methylphosphonate **2c**

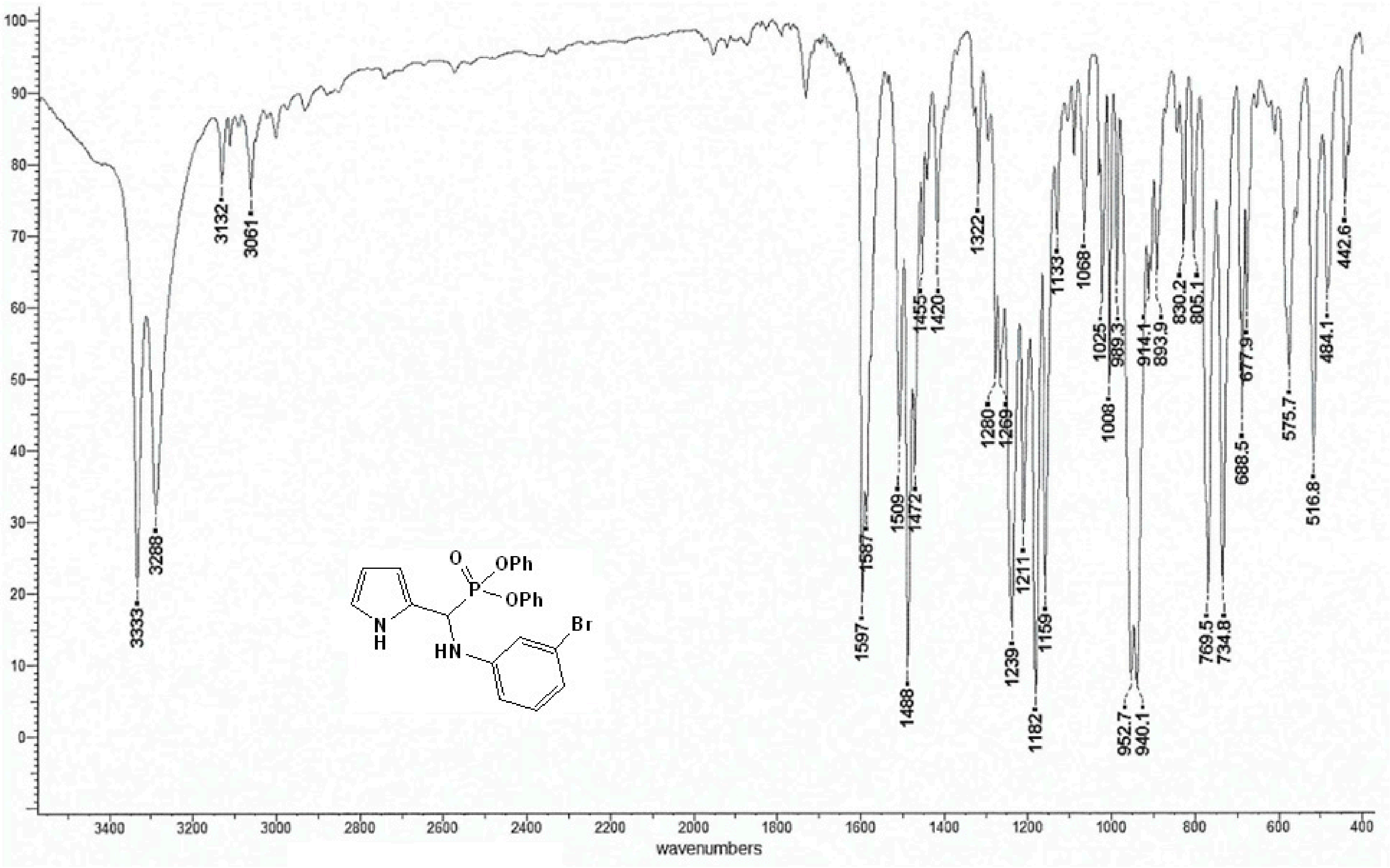

Figure S6d. FT-IR spectra of diphenyl N-(3-bromophenyl)-amino(pyrrol-2-yl)methylphosphonate **2d**

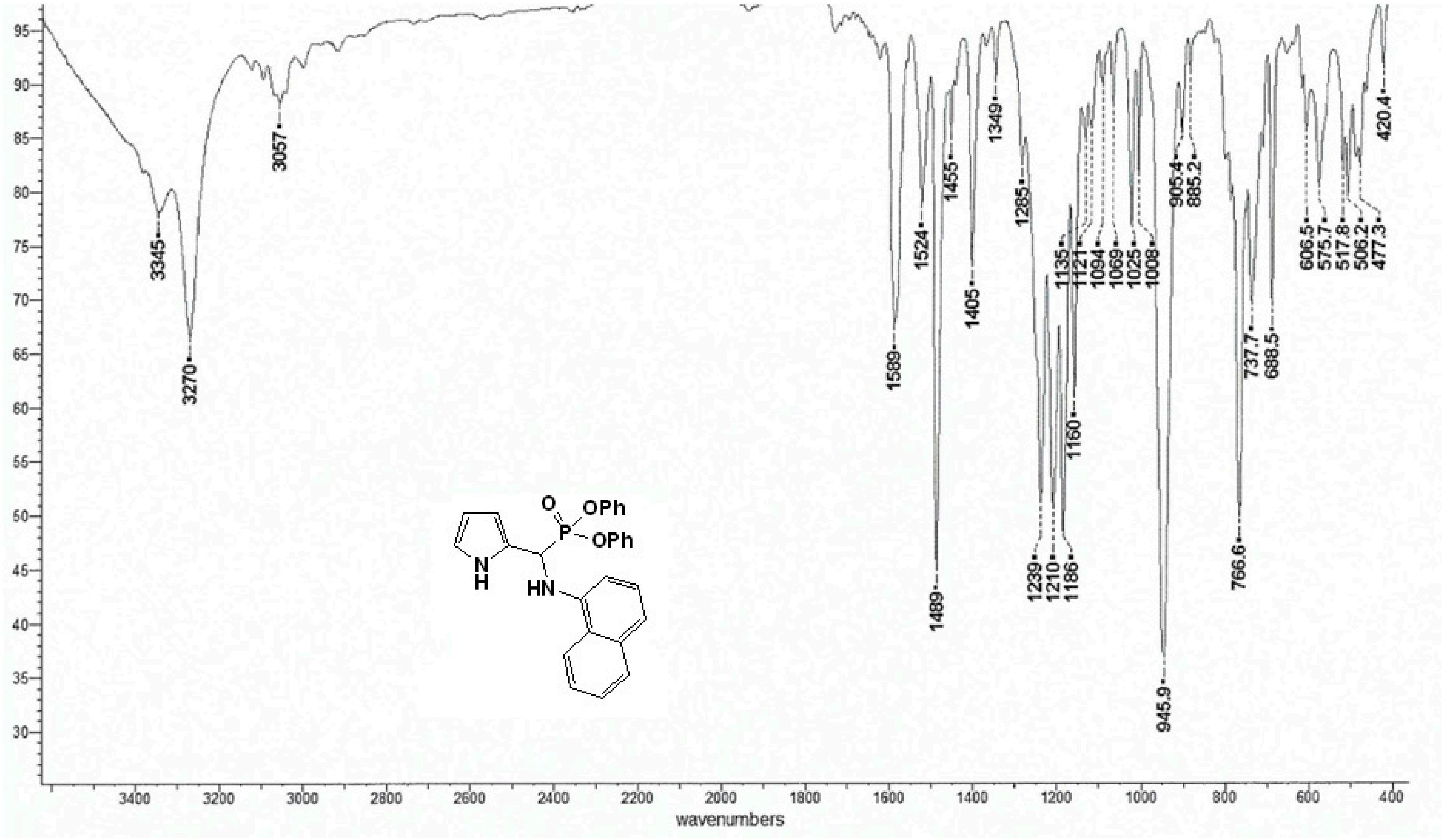

Figure S6e. FT-IR spectra of diphenyl N-(1-naphthyl)amino-(pyrrol-2-yl)methylphosphonate **2e**

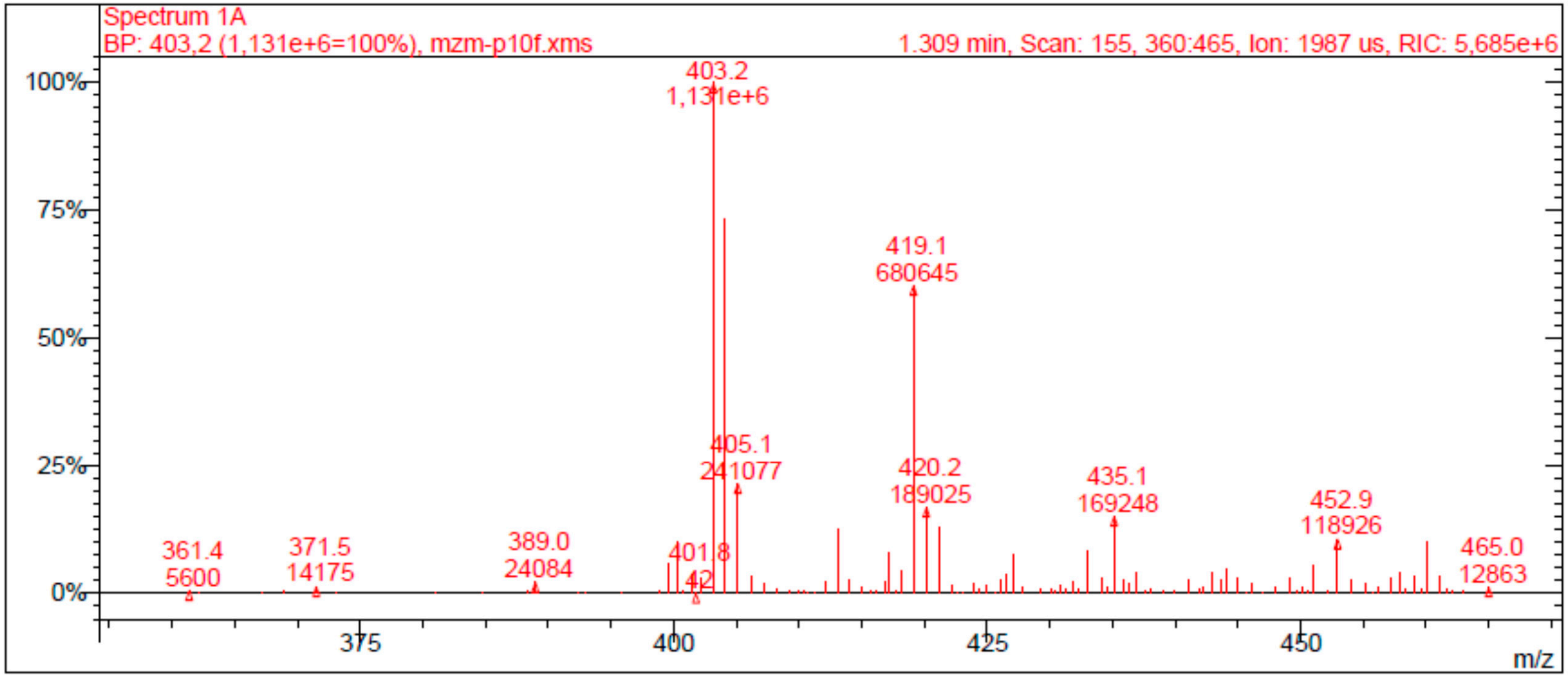

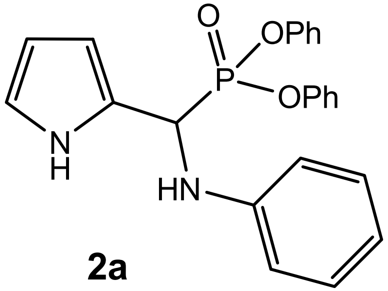

2a

Figure S7a. ESI-MS spectra of diphenyl N-phenylamino(pyrrol-2-yl)-methylphosphonate (**2a**).

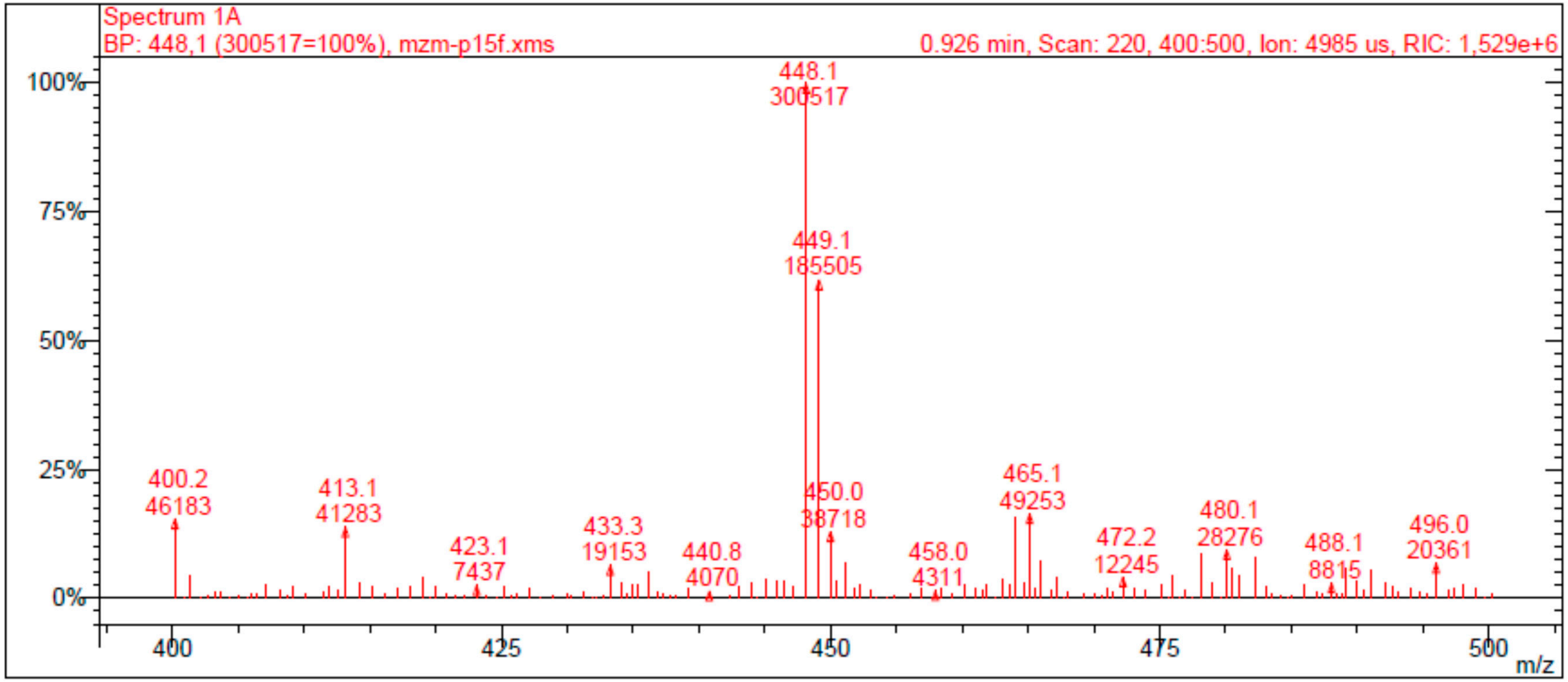

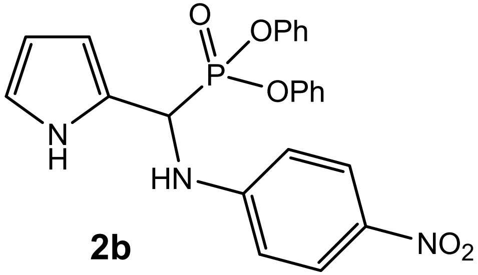

2b

Figure S7b. ESI-MS spectra of diphenyl *N*-(4-nitrophenyl)-amino(pyrrol-2-yl)methylphosphonate (**2b**).

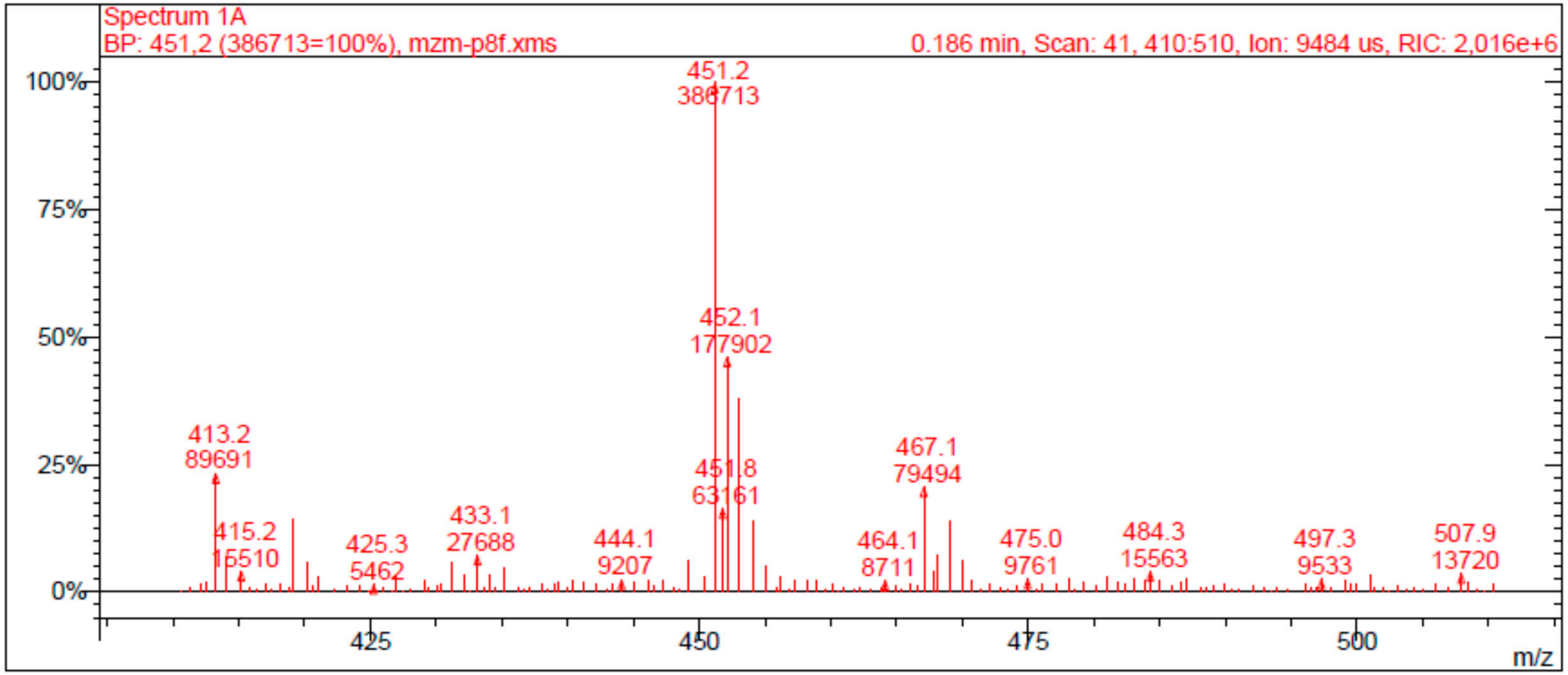

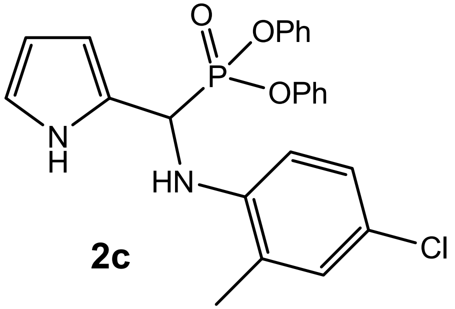

2c

Figure S7c. ESI-MS spectra of diphenyl N-(4-chloro-2-methylphenyl)amino-(pyrrol-2-yl)methylphosphonate (**2c**).

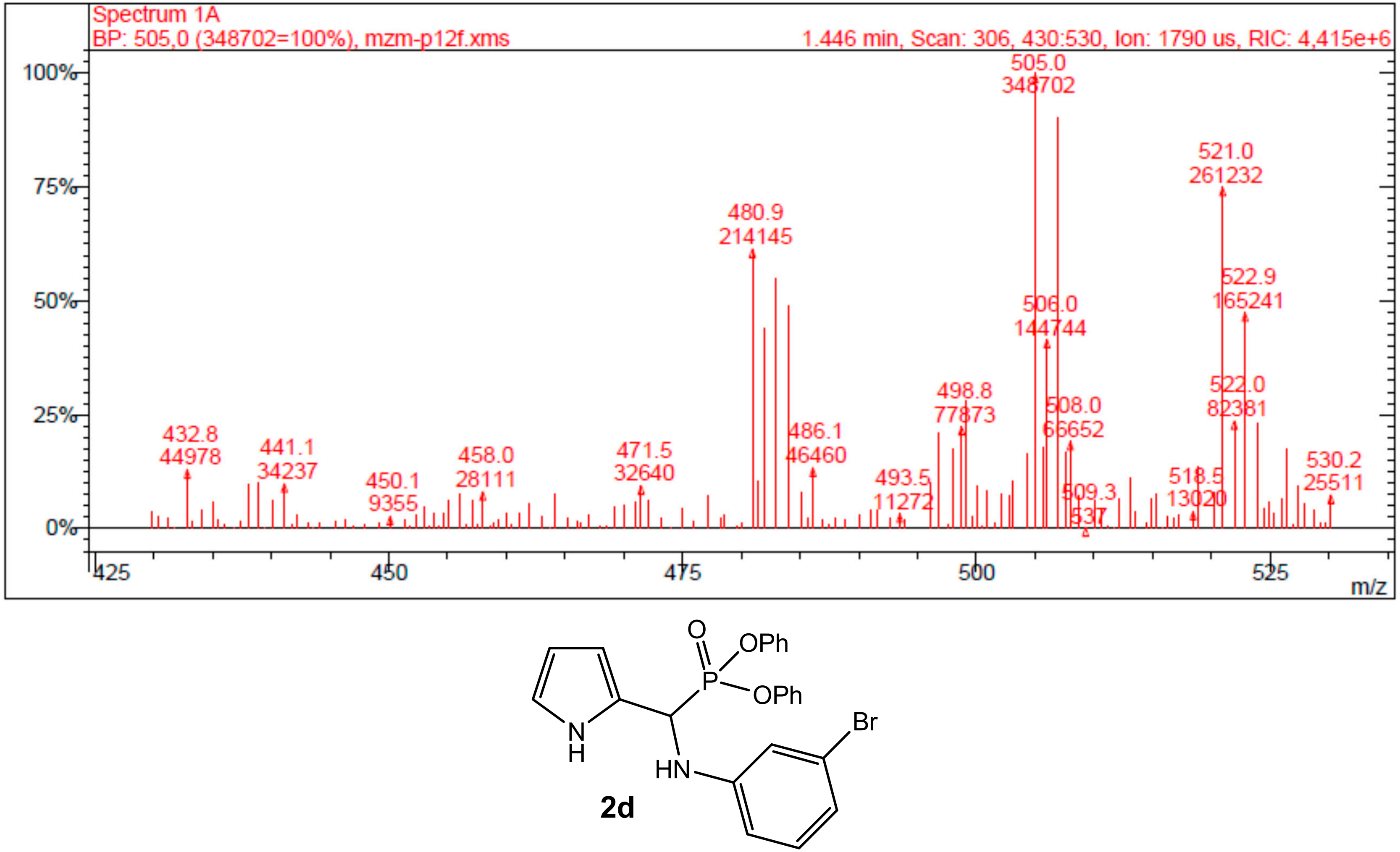

2d

Figure S7d. ESI-MS spectra of diphenyl *N*-(3-bromophenyl)-amino(pyrrol-2-yl)methylphosphonate (**2d**).

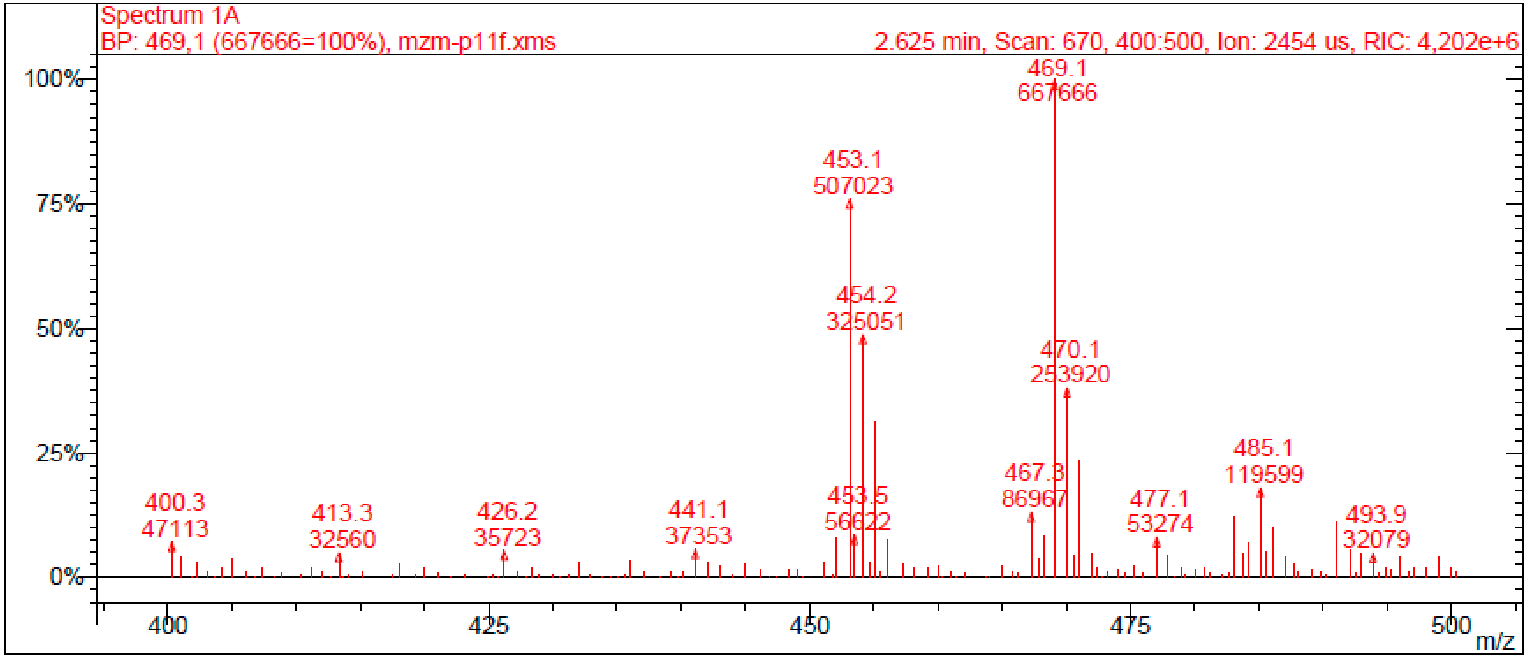

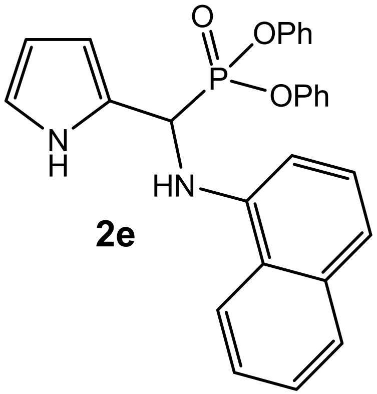

2e

Figure S7e. ESI-MS spectra of diphenyl *N*-(1-naphthyl)amino-(pyrrol-2-yl)methylphosphonate (**2e**).
